# The heterogeneous impact of targeted therapy on the prognosis of stage III/IV colorectal cancer patients with different subtypes of TP53 mutations

**DOI:** 10.1002/cam4.6766

**Published:** 2023-12-08

**Authors:** Jie Chen, Xiaona Chang, Xinyi Li, Jiaying Liu, Na Wang, Ying Wu, Liduan Zheng, Xiu Nie

**Affiliations:** ^1^ Department of Pathology, Wuhan Union Hospital, Tongji Medical College Huazhong University of Science and Technology Wuhan China

**Keywords:** colorectal neoplasms, genomic medicine, high‐throughput nucleotide sequencing, mutation, survival analysis

## Abstract

**Background:**

The relationship between molecular characteristics and the prognosis of colorectal cancer (CRC) patients has not been fully understood. This study explored the impact of targeted therapy on the prognosis of CRC patients with different TP53 mutations, in the context of comprehensive treatment.

**Methods:**

This study included patients with stage III/IV primary CRC from the electronic medical record system. TP53 mutations were detected via next‐generation sequencing (NGS) using formalin‐fixed paraffin‐embedded (FFPE) tissues. Applying two methods, we classified TP53 mutations as gain of function (GOF)/non‐GOF mutations or known/likely loss of function (LOF) mutations. Kaplan–Meier plot and parametric survival analysis were performed to evaluate the prognosis of CRC patients and identify potential predictors.

**Results:**

There were 286 patients included, of which 166 (58.04%) patients received targeted therapy and 120 (41.96%) did not. There were 286 patients in the TP53 GOF classification set and 247 in the TP53 LOF classification set. Parametric survival analysis, adjusted for sex, onset, KRAS mutation, sidedness, stage, and surgery, showed that receiving targeted therapy predicted better overall survival (OS) among patients who harbored TP53 GOF mutations (HR 0.40, 95% confidence interval (CI) [0.21, 0.76], *p* = 0.005) or known LOF mutations (HR 0.21, 95% CI [0.07, 0.60], *p* = 0.002). However, there was no significant impact of receiving targeted therapy on OS among patients harboring TP53 non‐GOF mutations (HR 1.68, 95% CI [0.50, 5.63], *p* = 0.403) or likely LOF mutations (HR 0.90, 95% CI [0.34, 2.39], *p* = 0.837).

**Conclusions:**

Receiving targeted therapy had a heterogeneous impact on the prognosis of CRC patients harboring different TP53 mutations. These results provide promising value for future personalized treatment and precision medicine.

## 1 BACKGROUND

Global cancer statistics by 2020 show that colorectal cancer (CRC) has the third highest incidence worldwide and is the second leading cause of cancer death.^1^ With the development of the social economy in developing countries, the high incidence region of CRC is no longer limited to developed countries such as Europe, North America, and Australia. In recent years, both CRC incidence and mortality have increased in East Asian populations.^1^, ^2^ The future increase in CRC cases would place a heavy burden on the healthcare system of each country, due to the poor quality of life and worse prognosis of CRC patients.^3^, ^4^

Colorectal cancer patients often show various responses to treatment, which may be due to the high tumor heterogeneity among different patients.^5^, ^6^ Previous studies have found significant differences in molecular characteristics between CRC patients with different tumor sites (right‐sided/left‐sided CRC) and ages of onset (early/late‐onset). For example, MSI‐H occurs more often in right‐sided CRCs, and early‐onset CRCs less frequently contain the BRAF V600E mutation.^3^, ^7^, ^8^ A recent study showed that the association of right‐sided CRC with poor prognosis depended on particular TP53 mutations.^9^ Several studies suggested that anti‐EGFR resistance may be associated with specific KRAS mutations but not with all KRAS mutations.^6^, ^10^ All the evidence above indicates that our understanding of the relevance between molecular features and clinicopathological characteristics as well as the prognosis of CRC patients is still limited.

Emerging studies have begun to classify mutations in TP53 and proposed the concept of oncomorphic/gain of function (GOF) and loss of function (LOF) mutations, both of which are tumor‐promoting due to distinct mechanisms.^11^, ^12^, ^13^ TP53 GOF mutations produce mutant p53 proteins with oncogenic functions, which are independent of wild‐type (WT) p53 functions.^11^, ^14^ TP53 LOF mutations lead to the loss of the DNA‐binding capability of mutant p53 proteins, similar to TP53 null status.^12^, ^15^ To date, there is no consensus on the classification criteria of TP53 mutations, and their impact on disease remains poorly understood.

Previous studies have shown that TP53 mutations could activate angiogenesis and promote tumor growth, which probably cause tumor cells to be more sensitive to anti‐VEGF/VEGFR treatment.^6^, ^16^, ^17^ Preclinical evidence demonstrated that VEGF and EGF share some common downstream signaling pathways to regulate cellular growth and proliferation.^18^, ^19^ And the RELAY trial showed benefit in prognosis of EGFR‐mutated metastatic non‐small‐cell lung cancer (NSCLC) patients who were treated with dual EGFR‐VEGF inhibition, comparing with those who were treated with placebo and anti‐EGFR therapy.^20^ However, TP53 mutation seemed to be a negative prognostic factor in EGFR‐mutated NSCLC patients treated by EGFR‐TKIs.^21^ Except for the different biological functions that may be caused by different TP53 mutations, in fact, in clinical practice, the situation was much more complex than controllable clinical trials, and patients may be undergoing multiple treatment regimens simultaneously: radiotherapy, chemotherapy, anti‐VEGF, and/or anti‐EGFR therapies, all of which could have an influence on patients' prognosis. Thus, by applying two methods for classification, this study explored the impact of targeted therapy on the prognosis of patients stratified by TP53 mutation status, under the comprehensive treatment of CRC.

## 2 METHODS

### 2.1 Study design

This retrospective cohort study included patients with stage III/IV primary colorectal cancer, who had been tested for TP53 mutation by next‐generation sequencing (NGS). The electronic medical record system of Union Hospital, Tongji Medical College, Huazhong University of Science and Technology was retrieved from January 2019 to August 2022 for eligible cases, using keywords “colon,” “rectum,” “colorectal,” “cancer,” “carcinoma,” “tumor,” “next‐generation sequencing,” and “NGS.” Patients with benign tumors, secondary colorectal cancers, or comorbidity with tumors at other sites were excluded from this study. This study was approved by the Ethics Committee of Wuhan Union Hospital (2018‐S377), and informed consent was obtained from patients.

Clinical data such as sex, age at diagnosis, primary tumor site (sidedness), stage, treatment, and partial follow‐up information were also collected from the electronic medical record system. Cases were defined as early‐onset CRC if patients' age at diagnosis was younger than 50 years old, otherwise, defined as late‐onset CRC. Primary tumors located in the ascending and transverse colon were called right‐sided CRC, while those located from the splenic flexure to rectum were called left‐sided CRC.^3^ The status of MSI or MMR was detected by NGS, PCR, or immunohistochemistry, respectively. The tumor stage of each patient mentioned in the subsequent analysis was evaluated by clinical doctors or pathologists at the initial diagnosis based on the eighth edition of the AJCC cancer staging system.^22^ Follow‐ups for the included patients were performed again in July 2023 to gather complete data.

All included patients received comprehensive treatment for CRC. Surgical treatment was performed for patients who were eligible for primary lesion resection. First‐line chemotherapy included FOLFOX, FOLFIRI, and CAPEOX in our center. Clinical doctors would choose or change different regimens according to patients' response and tolerance. Whether to add targeted agents into above regimens depended on the mutation status of gene and patient's own choice. Targeted therapy in this study included anti‐VEGF/VEGFR agent bevacizumab, fruquintinib, regorafenib, and anti‐EGFR agent cetuximab. Patients who received targeted therapy in this study received at least 6 cycles of intravenous targeted agents or had regularly oral administration of targeted agents.

### 2.2 Outcome

The primary outcome of this study was overall survival (OS). In this circumstance, each patient had a status of death or survival/censored. The survival time for OS was the period of time from diagnosis to death or censored. The secondary outcome was progression‐free survival (PFS). Progression was defined as death from any cause, metastases to other body sites, enlargement of the primary site, and new malignant lesions at the primary site. Cases would be labeled as progression or survival/censored in the case of PFS. The survival time for PFS was the period of time from diagnosis to progression or censored. The patient who was lost to follow‐up had a status as censored and survival time from diagnosis to the time of lost to follow‐up.

### 2.3 NGS test and TP53 mutation

Genomic DNA, extracted from formalin‐fixed paraffin‐embedded (FFPE) tissues of CRC patients in our center, was used in NGS test to detect the mutations of gene TP53, KRAS, and BRAF. There were two methods used to classify different TP53 mutations. One method classified TP53 mutations as GOF and non‐GOF mutations. In this GOF classification, missense mutations in DNA‐binding domain of p53 were defined as TP53 GOF mutations based on literature review.^9^, ^11^, ^12^, ^13^, ^14^ Missense mutations located in other regions of p53 except DNA‐binding domain, inframe deletion/insertion, and truncating mutations, such as nonsense, splicing, and frameshifts, were defined as TP53 non‐GOF mutations (Figure S1).

The other method classified TP53 mutations as known LOF and likely LOF mutations based on annotations in OncoKB database (https://www.oncokb.org),^23^ in which the mutation was annotated as known LOF when there was sufficient and reliable experimental evidence establishing the LOF function of this mutation, while the mutation was annotated as likely LOF if the evidence was limited or conflicting (Figure S1). When using this LOF classification, TP53 mutations not found or annotated as inconclusive in OncoKB were excluded from subsequent analysis.

### 2.4 Statistical analysis

Statistical differences in categorical variables were detected by the chi‐square (χ^2^) test or Fisher's exact test. Kaplan–Meier plots were generated to display survival data, and log‐rank test was used for between‐group comparisons. Parametric models based on Weibull distributions were performed for multivariate survival analysis as recommended, using WeibullReg() function in R package SurvRegCensCov (version 1.5).^24^, ^25^ Adjusted hazard ratios (HRs) with 95% confidence intervals (CIs) were calculated to show the impact of variables on OS and PFS. All statistical analyses were conducted by R version 4.2.1 (https://www.r‐project.org/). A two‐sided *p* value less than 0.05 was considered statistically significant.

## 3 RESULTS

### 3.1 Characteristics of patients

**FIGURE 1:**
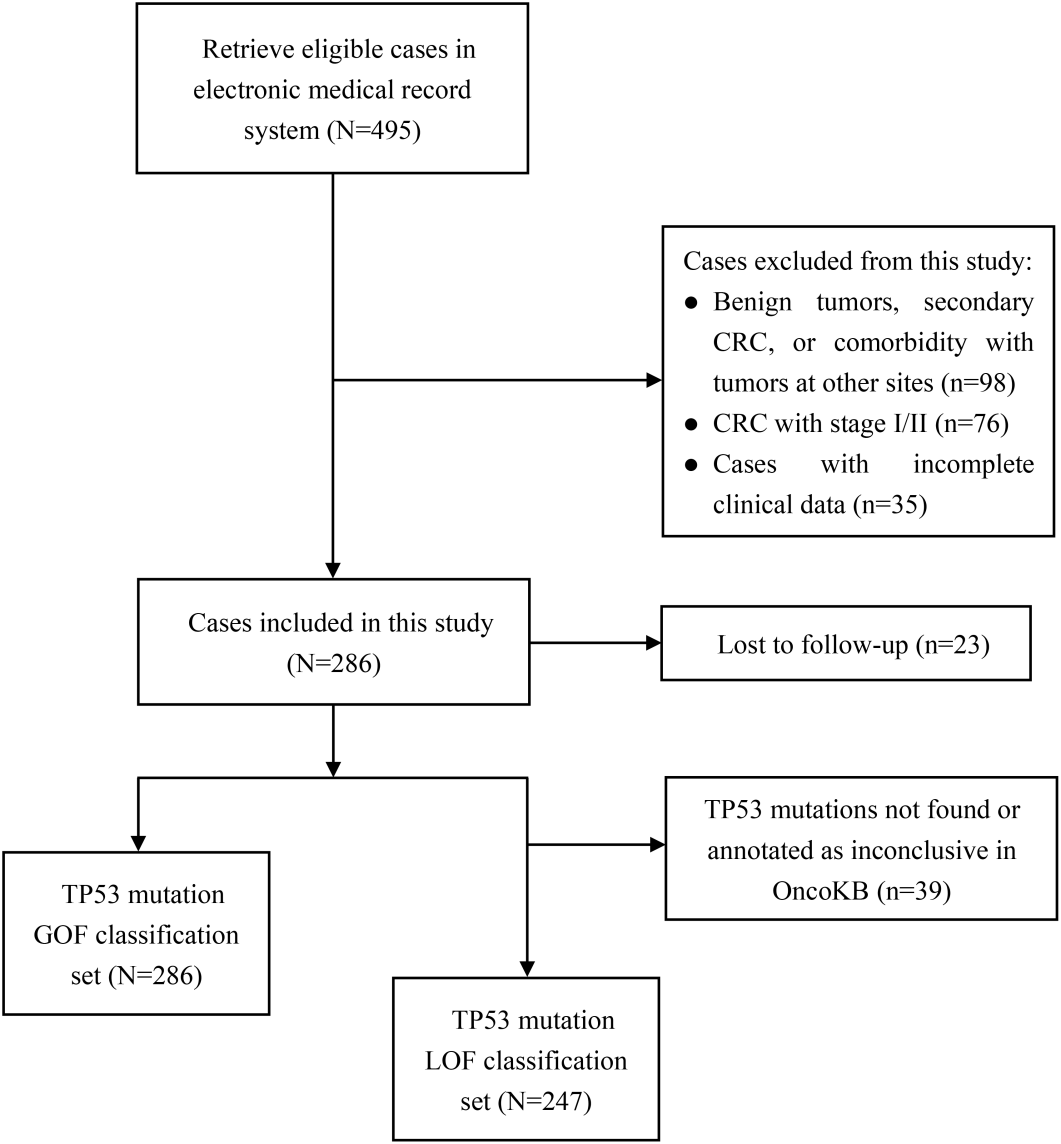
Flow chart of patient inclusion.

There were 495 cases retrieved from the electronic medical record system (Figure 1). Cases with benign tumors, secondary CRC, or comorbidity with tumors at other sites (*n* = 98), cases with CRC at stage I/II (*n* = 76), and cases with incomplete clinical data (*n* = 35) were excluded from this study. Finally, a total of 286 patients were included in this study with a median follow‐up period of 24.4 months, ranging from 1 month to 140.4 months. Twenty‐three patients who lost to follow‐up were also included in the final analysis set. There were 286 cases in the TP53 GOF classification set and 247 in the TP53 LOF classification set. In the GOF classification set, there were 90 (31.47%) patients with WT TP53, 65 (22.73%) with TP53 non‐GOF mutations, and 131 (45.80%) with TP53 GOF mutations (Table 1). The number of patients who received targeted therapy in this set was 166 (58.04%), while the number of patients who did not was 120 (41.96%) (Table S1). In the LOF classification set, there were 90 (36.43%) patients with WT TP53, 98 (39.68%) with TP53 likely LOF mutations, and 59 (23.89%) with TP53 known LOF mutations (Table 2). One hundred and forty‐two (57.49%) patients in this set received targeted therapy, while 105 (42.51%) did not (Table S1).

**TABLE 1:** Characteristics of colorectal cancer patients by the status of p53 Mutation based on gain of function classification (*N* = 286).

	TP53 WT (*n* = 90)	TP53 Mut (*n* = 196)	*p* Value	TP53 non‐GOF Mut (*n* = 65)	TP53 GOF Mut (*n* = 131)	*p* Value
Sex			0.351			0.409
Male	51 (56.67%)	124 (63.27%)		38 (58.46%)	86 (65.65%)	
Female	39 (43.33%)	72 (36.73%)		27 (41.54%)	45 (34.35%)	
Onset			0.154			0.561
Early onset	27 (30.00%)	42 (21.43%)		16 (24.62%)	26 (19.85%)	
Late onset	63 (70.00%)	154 (78.57%)		49 (75.38%)	105 (80.15%)	
Sidedness			0.035			0.682
Left‐sided	60 (66.67%)	155 (79.08%)		53 (81.54%)	102 (77.86%)	
Right‐sided	30 (33.33%)	41 (20.92%)		12 (18.46%)	29 (22.14%)	
Stage			0.094			0.543
III	47 (52.22%)	80 (40.82%)		29 (44.62%)	51 (38.93%)	
IV	43 (47.78%)	116 (59.18%)		36 (55.38%)	80 (61.07%)	
KRAS			0.252			0.917
WT	40 (44.44%)	103 (52.55%)		35 (53.85%)	68 (51.91%)	
Mut	50 (55.56%)	93 (47.45%)		30 (46.15%)	63 (48. 09%)	
BRAF			0.541			0.730
WT	81 (90.00%)	183 (93.37%)		61 (93.85%)	122 (93.13%)	
V600E mut	5 (5.56%)	7 (3.57%)		3 (4.61%)	4 (3.05%)	
Other mut	4 (4.44%)	6 (3.06%)		1 (1.54%)	5 (3.82)	
MSI/MMR			0.010			1.000
MSI‐H/dMMR	10 (11.11%)	5 (2.55%)		2 (3.08%)	3 (2.29%)	
MSS/pMMR	72 (80.00%)	177 (90.31%)		59 (90.77%)	118 (90.08%)	
Unknown	8 (8.89%)	14 (7.14%)		4 (6.15)	10 (7.63%)	
Surgery for primary lesion			0.574			0.215
No	23 (25.56%)	58 (29.59%)		15 (23.08%)	43 (32.82%)	
Yes	67 (74.44%)	138 (70.41%)		50 (76.92%)	88 (67.18%)	
Targeted therapy			0.853			0.387
No	38 (42.22%)	82 (41.84%)		24 (36.92%)	58 (44.28%)	
Anti‐VEGF/VEGFR	26 (28.89%)	61 (31.12%)		23 (35.38%)	38 (29.01%)	
Anti‐EGFR	8 (8.89%)	20 (10.21%)		7 (10.77%)	13 (9.92%)	
Anti‐VEGF/VEGFR + anti‐EGFR	7 (7.78%)	17 (8.67%)		8 (12.31%)	9 (6.87%)	
Yes (specific drugs unknown)	11 (12.22%)	16 (8.16%)		3 (4.62%)	13 (9.92%)	
Chemotherapy			1.000			0.400
No	3 (3.33%)	6 (3.06%)		3 (4.62%)	3 (2.29%)	
Yes	87 (96.67%)	190 (96.94%)		62 (95.38%)	128 (97.71%)	

Abbreviations: CRC, colorectal cancer; dMMR, deficient mismatch repair; GOF, gain of function; MSI‐H, high microsatellite instability level; MSS, microsatellite stable; pMMR, proficient mismatch repair; WT, wild‐type.

**TABLE 2:** Characteristics of colorectal cancer patients by the status of p53 Mutation based on loss of function classification (*N* = 247).

	TP53 WT (*n* = 90)	TP53 Mut (*n* = 157)	*p* Value	TP53 likely LOF Mut (*n* = 98)	TP53 known LOF Mut (*n* = 59)	*p* Value
Sex			0.247			1.000
Male	51 (56.67%)	102 (64.97%)		64 (65.31%)	38 (64.41%)	
Female	39 (43.33%)	55 (35.03%)		34 (34.69%)	21 (35.59%)	
Onset			0.337			0.875
Early onset	27 (30.00%)	37 (23.57%)		24 (24.49%)	13 (22.03%)	
Late onset	63 (70.00%)	120 (76.43%)		74 (75.51%)	46 (77.97%)	
Sidedness			0.006			1.000
Left‐sided	60 (66.67%)	130 (82.80%)		81 (82.65%)	49 (83.05%)	
Right‐sided	30 (33.33%)	27 (17.20%)		17 (17.35%)	10 (16.95%)	
Stage			0.188			1.000
III	47 (52.22%)	67 (42.68%)		42 (42.86%)	25 (42.37%)	
IV	43 (47.78%)	90 (57.32%)		56 (57.14%)	34 (57.63%)	
KRAS			0.296			0.821
WT	40 (44.44%)	82 (52.23%)		50 (51.02%)	32 (54.24%)	
Mut	50 (55.56%)	75 (47.77%)		48 (48.98%)	27 (45.76%)	
BRAF			0.253			1.000
WT	81 (90.00%)	149 (94.90%)		93 (94.90%)	56 (94.92%)	
V600E mut	5 (5.56%)	3 (1.91%)		2 (2.04%)	1 (1.69%)	
Other mut	4 (4.44%)	5 (3.19%)		3 (3.06%)	2 (3.39%)	
MSI/MMR			0.017			0.501
MSI‐H/dMMR	10 (11.11%)	4 (2.55%)		2 (2.04%)	2 (3.39%)	
MSS/pMMR	72 (80.00%)	141 (89.81%)		90 (91.84%)	51 (86.44%)	
Unknown	8 (8.89%)	12 (7.64%)		6 (6.12%)	6 (10.17%)	
Surgery for primary lesion			0.629			0.660
No	23 (25.56%)	46 (29.30%)		27 (27.55%)	19 (32.20%)	
Yes	67 (74.44%)	111 (70.70%)		71 (72.45%)	40 (67.80%)	
Targeted therapy			0.825			0.097
No	38 (42.22%)	67 (42.68%)		34 (34.69%)	33 (55.93%)	
Anti‐VEGF/VEGFR	26 (28.89%)	49 (31.21%)		35 (35.71%)	14 (23.73%)	
Anti‐EGFR	8 (8.89%)	16 (10.19%)		10 (10.21%)	6 (10.17%)	
Anti‐VEGF/VEGFR + anti‐EGFR	7 (7.78%)	13 (8.28%)		9 (9.18%)	4 (6.78%)	
Yes (specific drugs unknown)	11 (12.22%)	12 (7.64%)		10 (10.21%)	2 (3.39%)	
Chemotherapy			0.708			1.000
No	3 (3.33%)	4 (2.55%)		3 (3.06%)	1 (1.69%)	
Yes	87 (96.67%)	153 (97.45%)		95 (96.94%)	58 (98.31%)	

Abbreviations: CRC, colorectal cancer; dMMR, deficient mismatch repair; GOF, gain of function; MSI‐H, high microsatellite instability level; MSS, microsatellite stable; pMMR, proficient mismatch repair; WT, wild‐type.

Among all patients, the mutation rates of gene TP53 and KRAS were 68.53% (196/286) and 50.00% (143/286), respectively. BRAF mutations were detected in 7.69% (22/286) of patients, in which the BRAF V600E mutation accounts for 4.20% (12/286). Tumor tissues were MSI‐H/dMMR in only 5.24% (15/286) of patients. Almost all of the patients, 96.50% (276/286) received chemotherapy. Given the small number of cases with MSI‐H/dMMR, BRAF mutation and not receiving chemotherapy, these covariates were not included in subsequent multivariate analyses. Other characteristics of patients, such as sex, sidedness, stage, etc., were also shown in Table 1 and Table 2.

### 3.2 Prognosis of patients in TP53 mutation GOF classification set

**FIGURE 2:**
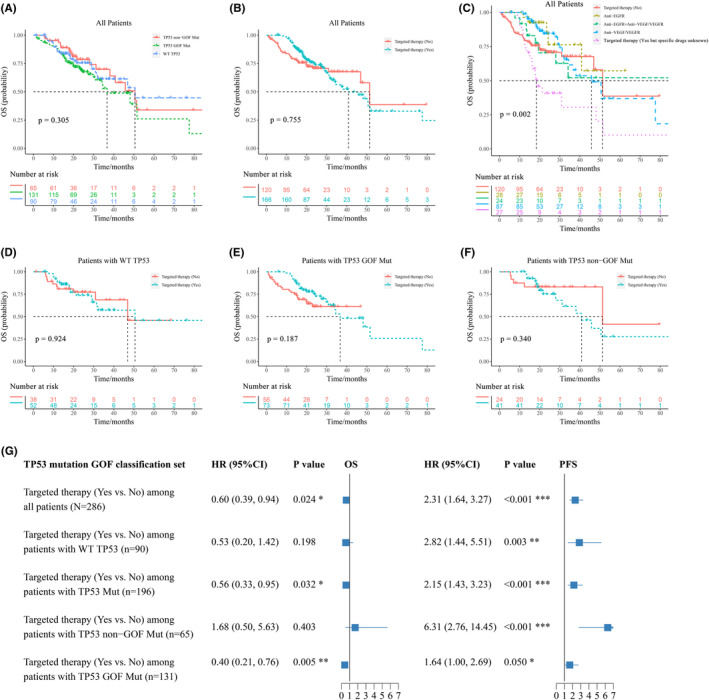
Prognosis of patients in TP53 mutation GOF classification set. (A) to (F), overall survival (OS) of patients with different statuses of TP53 mutations and targeted therapy; (G) multivariate parametric survival analysis of OS and PFS in TP53 mutation GOF classification set, adjusted for sex, onset, KRAS mutation, sidedness, stage, and surgery.

In the GOF classification set, the median OS for patients with WT TP53, TP53 GOF mutations, and TP53 non‐GOF mutations was 50.57 months, 36.67 months, and 50.50 months, respectively (Figure 2A). The median OS was 40.80 months and 51.33 months, for patients who received targeted therapy and those who did not (Figure 2B). Patients who were treated with anti‐VEGF/VEGFR had a median OS of 45.73 months, while the median OS for patients who were treated with anti‐EGFR was not observed (Figure 2C). Among patients with WT TP53, the median OS was 50.57 months and 46.90 months for those who received targeted therapy and those who did not (Figure 2D). Among patients who harbored TP53 GOF mutations, the median OS for patients who received targeted therapy was 36.67 months, while the median OS for those who did not was not observed (Figure 2E). Among patients who harbored TP53 non‐GOF mutations, the median OS was 40.80 months and 51.33 months for those who received targeted therapy and those who did not (Figure 2F).

The median PFS was 18.30 months, 14.97 months, and 16.27 months, for patients with WT TP53, TP53 GOF mutations, and TP53 non‐GOF mutations, respectively (Figure S2A). The median PFS for patients who received targeted therapy was 13.47 months, while it was 32.03 months for patients who did not (Figure S2B). Patients who were treated with anti‐VEGF/VEGFR had a median PFS of 13.90 months, while that was 13.53 months in patients who were treated with anti‐EGFR (Figure S2C). Among patients containing WT TP53, the median PFS was 13.97 months and 38.17 months for those who received targeted therapy and those who did not (Figure S2D). Among patients who harbored TP53 GOF mutations, the median PFS was 12.03 months and 32.03 months for those who received targeted therapy and those who did not (Figure S2E). Among patients who harbored TP53 non‐GOF mutations, the median PFS for patients who received targeted therapy was 13.33 months, while the median PFS for those who did not was not observed (Figure S2F).

Multivariate parametric survival analysis, adjusted for sex, onset, KRAS mutation, sidedness, stage, and surgery, showed that patients who received targeted therapy had better OS compared with those who did not, among all patients (HR 0.60, 95% confidence interval (CI) [0.39, 0.94], *p* = 0.024) or in patients with TP53 mutations (HR 0.56, 95% CI [0.33, 0.95], *p* = 0.032), while there was no statistical difference among patients with WT TP53 (HR 0.53, 95% CI [0.20, 1.42], *p* = 0.198) (Figure 2G, Table S2). Receiving targeted therapy predicted better OS among patients who harbored TP53 GOF mutations (HR 0.40, 95% CI [0.21, 0.76], *p* = 0.005), whereas not among patients who harbored TP53 non‐GOF mutations (HR 1.68, 95% CI [0.50, 5.63], *p* = 0.403) (Figure 2G, Table S2). However, receiving targeted therapy was associated with poorer PFS regardless of the mutation status of TP53.

### 3.3 Prognosis of patients in TP53 mutation LOF classification set

**FIGURE 3:**
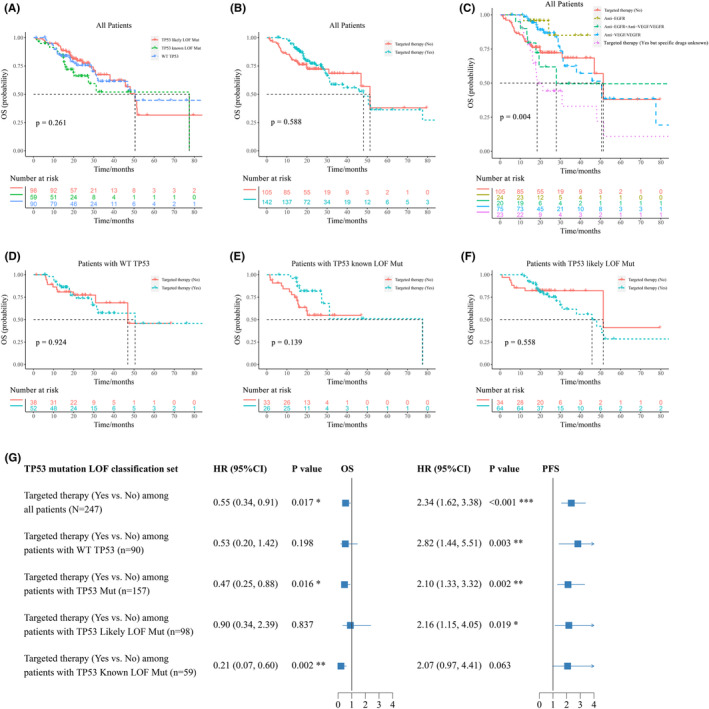
Prognosis of patients in TP53 mutation LOF classification set. (A) to (F), overall survival (OS) of patients with different statuses of TP53 mutations and targeted therapy; (G) multivariate parametric survival analysis of OS and PFS in TP53 mutation LOF classification set, adjusted for sex, onset, KRAS mutation, sidedness, stage, and surgery.

In the LOF classification set, the median OS for patients with WT TP53, TP53 known LOF mutations, and TP53 likely LOF mutations was 50.57 months, 77.53 months, and 50.50 months, respectively (Figure 3A). The median OS for patients who received targeted therapy was 48.10 months, while the median OS for patients who did not was 51.33 months (Figure 3B). Patients who were treated with anti‐VEGF/VEGFR had a median OS of 50.50 months, while the median OS for patients who were treated with anti‐EGFR was not observed (Figure 3C). Among patients with WT TP53, the median OS was 50.57 months and 46.90 months for those who received targeted therapy and those who did not (Figure 3D). Among patients who harbored TP53 known LOF mutations, the median OS for patients who received targeted therapy was 77.53 months, while the median OS for those who did not was not observed (Figure 3E). Among patients who harbored TP53 likely LOF mutations, the median OS was 45.73 months and 51.33 months for those who received targeted therapy and those who did not (Figure 3F).

The median PFS was 18.30 months, 14.97 months, and 15.87 months, for patients with WT TP53, TP53 known LOF mutations, and TP53 likely LOF mutations, respectively (Figure S3A). The median PFS was 13.63 months for patients who received targeted therapy and 30.90 months for those who did not (Figure S3B). Patients who were treated with anti‐VEGF/VEGFR had a median PFS of 13.97 months, while that was 13.53 months in patients who were treated with anti‐EGFR (Figure S3C). Among patients containing WT TP53, the median PFS was 13.97 months and 38.17 months for those who received targeted therapy and those who did not (Figure S3D). Among patients who harbored TP53 known LOF mutations, the median PFS for patients who received targeted therapy was 11.63 months, while the median PFS for those who did not was not observed (Figure S3E). Among patients who harbored TP53 likely LOF mutations, the median PFS was 13.58 months and 27.03 months for those who received targeted therapy and those who did not (Figure S3F).

Multivariate survival analysis based on parametric models, adjusted for sex, onset, KRAS mutation, sidedness, stage, and surgery, showed that patients who received targeted therapy had a better OS than those who did not, among all patients (HR 0.55, 95% CI [0.34, 0.91], *p* = 0.017) or in patients with TP53 mutations (HR 0.47, 95% CI [0.25, 0.88], *p* = 0.016), while there was no statistical difference among patients with WT TP53 (HR 0.53, 95% CI [0.20, 1.42], *p* = 0.198) (Figure 3G, Table S3). Among patients with TP53 known LOF mutations, receiving targeted therapy predicted better OS compared with not receiving targeted therapy (HR 0.21, 95% CI [0.07, 0.60], *p* = 0.002), while among patients with TP53 likely LOF mutations, there was no statistical significance between patients who received targeted therapy and who did not (HR 0.90, 95% CI [0.34, 2.39], *p* = 0.837) (Figure 3G, Table S3). However, receiving targeted therapy was associated with poorer PFS regardless of the mutation status of TP53.

### 3.4 Overall survival of patients with progression

**FIGURE 4:**
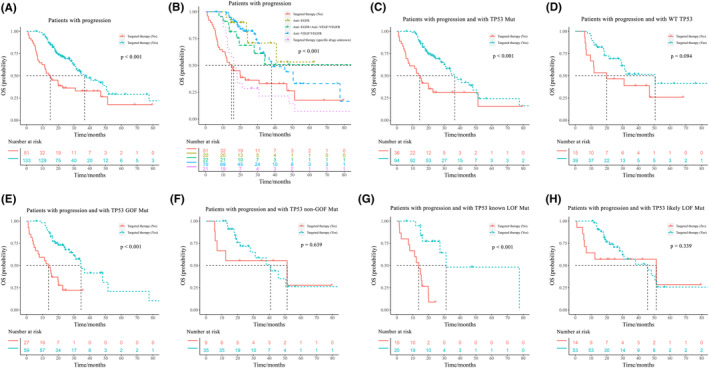
Overall survival of patients with progression. Overall survival (OS) of patients who received targeted therapy or not among patients with progression (A, B), patients with progression and harboring TP53 mutations (C), patients with progression and harboring WT TP53 (D), patients with progression and harboring TP53 GOF mutations (E) or non‐GOF mutations (F), patients with progression and harboring TP53 known LOF mutations (G) or likely LOF mutations (H).

There were totally 184 patients experienced the progression of disease, 130 of whom harbored TP53 mutations, while 54 with WT TP53. Among patients with progression, the median OS for patients who received targeted therapy was 36.67 months, while the median OS for patients who did not was 14.97 months (*p* < 0.001) (Figure 4A). Patients who were treated with anti‐VEGF/VEGFR had a median OS of 38.07 months, while the median OS for patients who were treated with anti‐EGFR was not observed (Figure 4B). In patients with progression and harboring TP53 mutations, those who received targeted therapy had a longer median OS than those who did not (36.67 months vs. 14.40 months, *p* < 0.001) (Figure 4C), whereas in patients with progression and with WT TP53, there was no statistical significance between the median OS of patients who received targeted therapy and who did not (50.57 months vs. 19.90 months, *p* = 0.094) (Figure 4D). Receiving targeted therapy prolonged the median OS of patients who experienced progression and with TP53 GOF mutations (34.47 months vs. 13.83 months, *p* < 0.001) (Figure 4E). However, whether receiving targeted therapy or not had almost no impact on patients who experienced progression and with TP53 non‐GOF mutations (40.80 months vs. 51.33 months, *p* = 0.639) (Figure 4F). Similarly, receiving targeted therapy prolonged the median OS of patients who experienced progression and with TP53 known LOF mutations (31.17 months vs. 13.83 months, *p* < 0.001) (Figure 4G), while as for patients who experienced progression and with TP53 likely LOF mutations, there was no significant difference between the median OS of those who received targeted therapy and those who did not (45.73 months vs. 51.33 months, *p* = 0.339) (Figure 4H).

## 4 DISCUSSION

In the GOF classification set, targeted therapy independently predicted better OS in patients with WT TP53 and patients with TP53 GOF mutations, but not in patients with TP53 mutations or patients with TP53 non‐GOF mutations. In the LOF classification set, targeted therapy independently predicted better OS in patients with WT TP53, patients with TP53 mutations, and patients with TP53 known LOF mutations, but not in patients with likely LOF mutations. Consistent with previous studies, different classifications for TP53 mutations have little impact on the final results.^13^ Through independent analysis of these two classification sets, similar results were obtained that the impact of targeted therapy on OS was heterogeneous among CRC patients with TP53 mutations, in the context of comprehensive treatment. In other words, targeted therapy may benefit the OS of partial CRC patients with specific TP53 mutations (GOF or known LOF mutations), whereas probably has no significant impact on the OS of CRC patients with other TP53 mutations (non‐GOF or likely LOF mutations).

The tumor suppressor gene TP53 is widely involved in various biological processes to prevent tumorigenesis.^26^, ^27^ And TP53 mutation is associated with poor prognosis in most cancers, such as breast cancer, lung cancer, hematopoietic cancers, as well as CRC.^28^, ^29^, ^30^, ^31^ This was in line with our results that CRC patients with TP53 mutations had worse PFS than those with WT TP53. Mutp53 could reprogram cell metabolism, make cancer cells adapt to various stresses, and facilitate tumor cell migration, which all together promote tumor survival.^32^, ^33^ However, the relationship between specific TP53 mutations and the prognosis of tumor patients is still unclear.^27^ Although without significant differences, this study explored the impact of particular subtypes of TP53 mutations on the prognosis of CRC patients stratified by the status of targeted therapy, providing limited evidence to this field.

The most common agents used in targeted therapy for CRC patients were anti‐EGFR (e.g., cetuximab) and anti‐VEGF/VEGFR agents (e.g., bevacizumab).^3^, ^6^ In order to rule out confounding effects on treatment choices, KRAS mutation was also adjusted in multivariate survival analysis. The interactions between molecular features of CRC and treatment response are complicated. Despite plenty of studies investigating the effectiveness of targeted therapy, there were still no suitable biomarkers to predict the anti‐tumor activity of anti‐EGFR or anti‐VEGF/VEGFR agents.^34^, ^35^ Consensus molecular subtypes (CMS) classification for CRC cannot provide more detailed guidance for the selection of targeted therapy either.^36^ The recent conception of anti‐EGFR therapy rechallenge implied the mechanism of resistance or sensitivity to anti‐EGFR drugs in the same patient, as the proportions of RAS WT clones and RAS‐mutant clones in CRC might be changed dynamically during the treatment period.^34^, ^37^

Although there were no officially approved anti‐cancer drugs targeting mutp53,^26^ TP53 mutations do have an impact on the effectiveness of targeted therapy in tumor patients. Two meta‐analyses showed that TP53 co‐mutation was a negative prognostic factor in lung cancer patients who were with EGFR mutations and were treated with targeted therapy, whereas there was no predictive value in patients who were treated with non‐targeted therapy.^38^, ^39^ Studies in patients with diverse cancers showed that TP53 mutations could predict well response to anti‐VEGF therapy.^19^, ^40^ However, none have investigated the impact of different subtypes of TP53 mutations on the prognosis of cancer patients receiving targeted therapy. This study indicated the heterogeneous impact of targeted therapy on the prognosis of CRC patients who harbored TP53 GOF/known LOF and non‐GOF/likely LOF mutations. The possible reason for this phenomenon may be that different subtypes of TP53 mutations display various cell functions due to different structures or stabilities of mutant proteins, leading to distinct responses to targeted therapy.^27^, ^41^ More research is needed to investigate the specific biological functions of various subtypes of TP53 mutations before we can reveal the mechanism of heterogeneous response to targeted therapy in patients with different subtypes of TP53 mutations.

Besides, there are also some inconsistencies in the results of this study. The most notable is that targeted therapy benefits the OS of CRC patients, while seems to be a risk factor for PFS. This may be because there were more stage IV patients who were more likely to undergo progression among patients who received targeted therapy than those who did not. Moreover, there might be other possible factors that could affect the patient's choice of targeted therapy, such as financial status.^42^, ^43^ As mentioned in the *Characteristics of patients*, almost all of the patients in this study received chemotherapy. To our knowledge, lots of CRC patients would not choose targeted therapy until they respond poorly to chemotherapy, or even have suffered from progression under routine chemotherapy, since NGS tests and drugs for targeted therapy are much more expensive than routine chemotherapy.^6^, ^44^ However, receiving targeted therapy could still benefit OS in patients with progression (Figure 4).

This is the first study to explore the impact of targeted therapy on the prognosis of CRC patients stratified by different subgroups of TP53 mutations, till now. The conclusion drawn from this study, that targeted therapy benefits OS of CRC patients with specific TP53 mutations but not all TP53 mutations, was reliable and robust, given that analysis in two classification sets yielded similar results. There are also some limitations. Firstly, selective bias may exist for this study with a not large sample size. In addition, since there are currently no consensus classification criteria for TP53 mutations, we are not able to precisely distinguish TP53 mutations with different functions. Thus, potential intragroup heterogeneity may also have an impact on the final results.

## 5 CONCLUSIONS

This study showed that under the comprehensive treatment of CRC, receiving targeted therapy predicted better OS in patients who harbored TP53 GOF / known LOF mutations, but not in patients with TP53 non‐GOF / likely LOF mutations, providing evidence for the application of future personalized and precision medicine in the management of CRC patients.

## AUTHOR CONTRIBUTIONS

**Jie Chen:** Conceptualization (supporting); data curation (lead); formal analysis (lead); investigation (equal); methodology (equal); resources (lead); software (lead); visualization (lead); writing – original draft (lead); writing – review and editing (equal). **Xiaona Chang:** Conceptualization (equal); data curation (supporting); formal analysis (supporting); investigation (equal); methodology (equal); supervision (equal); validation (supporting); visualization (supporting); writing – review and editing (equal). **Xinyi Li:** Resources (supporting). **Jiaying Liu:** Resources (supporting). **Na Wang:** Resources (supporting). **Ying Wu:** Resources (supporting). **Liduan Zheng:** Conceptualization (equal); methodology (equal); supervision (equal); validation (lead); writing – review and editing (supporting). **Xiu Nie:** Conceptualization (supporting); validation (supporting); writing – review and editing (supporting).

## FUNDING INFORMATION

This research received no external funding.

## CONFLICT OF INTEREST STATEMENT

All authors declare that they have no conflict of interest.

## ETHICS STATEMENT

This study was approved by the Ethics Committee of Wuhan Union Hospital (2018‐S377).

## PATIENT CONSENT

Informed consent was obtained from patients.

## Supporting information


Data S1.

Tables S1–S3.

Figure S1.

Figure S2.

Figure S3.

Data S2.

## Data Availability

The data that supports the findings of this study are available in supplementary data.
