# Supplementary material for: The heterogeneous impact of targeted therapy on the prognosis of stage III/IV colorectal cancer patients with different subtypes of TP53 mutations
Source: Cancer Med. 2023 Dec 8;12(24):21920–32. doi: 10.1002/cam4.6766 (PMC10757131; doi:10.1002/cam4.6766)
Supplement: Supplementary file 2 — Tables S1–S3. [file CAM4-12-21920-s002.docx]

**Supplementary Table 1. Characteristics of CRC patients by the status of targeted therapy**

|  | GOF set (n=286) | | P value | LOF set (n=247) | | P value |
| --- | --- | --- | --- | --- | --- | --- |
| Targeted therapy | **(No) (n=120)** | **(Yes) (n=166)** |  | **(No) (n=105)** | **(Yes) (n=142)** |  |
| Sex |  |  | 1.000 |  |  | 0.886 |
| Male | 73 (60.83%) | 102 (61.45%) |  | 64 (60.95%) | 89 (62.68%) |  |
| Female | 47 (39.17%) | 64 (38.55%) |  | 41 (39.05%) | 53 (37.32%) |  |
| Onset |  |  | 1.000 |  |  | 0.836 |
| Early onset | 29 (24.17%) | 40 (24.10%) |  | 26 (24.76%) | 38 (26.76%) |  |
| Late onset | 91 (75.83%) | 126 (75.90%) |  | 79 (75.24%) | 104 (73.24%) |  |
| Sidedness |  |  | 0.081 |  |  | 0.148 |
| Left-sided | 97 (80.83%) | 118 (71.08%) |  | 86 (81.90%) | 104 (73.24%) |  |
| Right-sided | 23 (19.17%) | 48 (28.92%) |  | 19 (18.10%) | 38 (26.76%) |  |
| Stage |  |  | <0.001 |  |  | <0.001 |
| III | 75 (62.50%) | 52 (31.33%) |  | 67 (63.81%) | 47 (33.10%) |  |
| IV | 45 (37.50%) | 114 (68.67%) |  | 38 (36.19%) | 95 (66.90%) |  |
| KRAS |  |  | 0.719 |  |  | 1.000 |
| WT | 62 (51.67%) | 81 (48.80%) |  | 52 (49.52%) | 70 (49.30%) |  |
| Mut | 58 (48.33%) | 85 (51.20%) |  | 53 (50.48%) | 72 (50.70%) |  |
| TP53 |  |  | 1.000 |  |  | 1.000 |
| WT | 38 (31.67%) | 52 (31.33%) |  | 38 (36.19%) | 52 (36.62%) |  |
| Mut | 82 (68.33%) | 114 (68.67%) |  | 67 (63.81%) | 90 (63.38%) |  |
| BRAF |  |  | 0.484 |  |  | 0.746 |
| WT | 112 (93.33%) | 152 (91.57%) |  | 97 (92.38%) | 133 (93.66%) |  |
| V600E mut | 3 (2.50%) | 9 (5.42%) |  | 3 (2.86%) | 5 (3.52%) |  |
| Other mut | 5 (4.17%) | 5 (3.01%) |  | 5 (4.76%) | 4 (2.82%) |  |
| MSI/MMR |  |  | <0.001 |  |  | <0.001 |
| MSI-H/dMMR | 14 (11.67%) | 1 (0.60%) |  | 13 (12.38%) | 1 (0.70%) |  |
| MSS/pMMR | 96 (80.00%) | 153 (92.17%) |  | 82 (78.10%) | 131 (92.26%) |  |
| unknown | 10 (8.33%) | 12 (7.23%) |  | 10 (9.52%) | 10 (7.04%) |  |
| Surgery for primary lesion | | | 0.145 |  |  | 0.050 |
| No | 28 (23.33%) | 53 (31.93%) |  | 22 (20.95%) | 47 (33.10%) |  |
| Yes | 92 (76.67%) | 113 (68.07%) |  | 83 (79.05%) | 95 (66.90%) |  |
| Chemotherapy |  |  | 0.005 |  |  | 0.044 |
| No | 8 (6.67%) | 1 (0.60%) |  | 6 (5.71%) | 1 (0.70%) |  |
| Yes | 112 (93.33%) | 165 (99.40%) |  | 99 (94.29%) | 141 (99.30%) |  |

Abbreviation: CRC, colorectal cancer; WT, wild-type; MSI-H, high microsatellite instability level; MSS, microsatellite stable; dMMR, deficient mismatch repair; pMMR, proficient mismatch repair.

**Supplementary Table 2.** **Parametric models for survival analysis by statuses of p53 Mutation based on GOF classification (N=286)**

|  | OS | | PFS | |
| --- | --- | --- | --- | --- |
|  | **HR (95%CI)** | **P value** | **HR (95%CI)** | **P value** |
| All patients (N=286) |  |  |  |  |
| Male (vs. Female) | 1.18 (0.76, 1.85) | 0.450 | 1.10 (0.80, 1.50) | 0.559 |
| Late onset (vs. Early onset) | 1.97 (1.08, 3.61) | 0.027 | 1.00 (0.70, 1.43) | 0.993 |
| KRAS Mut (vs. WT) | 1.24 (0.80, 1.93) | 0.330 | 1.42 (1.04, 1.94) | 0.025 |
| TP53 Mut (vs. WT) | 1.07 (0.66, 1.72) | 0.784 | 1.30 (0.94, 1.81) | 0.115 |
| Right-sided (vs. Left-sided) | 1.42 (0.88, 2.28) | 0.149 | 1.00 (0.70, 1.42) | 0.989 |
| Stage IV (vs. Stage III) | 1.71 (1.03, 2.84) | 0.039 | 1.03 (0.72, 1.48) | 0.851 |
| Surgery for primary lesion (Yes vs. No) | 0.21 (0.13, 0.34) | <0.001 | 0.57 (0.39, 0.82) | 0.002 |
| Targeted therapy (Yes vs. No) | 0.60 (0.39, 0.94) | 0.024 | 2.31 (1.64, 3.27) | <0.001 |
| Patients with WT TP53 (n=90) | | | | |
| Male (vs. Female) | 1.19 (0.50, 2.87) | 0.690 | 1.90 (1.06, 3.40) | 0.028 |
| Late onset (vs. Early onset) | 4.25 (1.26, 14.35) | 0.019 | 1.01 (0.54, 1.89) | 0.977 |
| KRAS Mut (vs. WT) | 1.31 (0.54, 3.13) | 0.545 | 1.09 (0.61, 1.96) | 0.760 |
| Right-sided (vs. Left-sided) | 1.10 (0.45, 2.68) | 0.834 | 1.39 (0.73, 2.65) | 0.310 |
| Stage IV (vs. Stage III) | 1.73 (0.64, 4.74) | 0.277 | 0.64 (0.33, 1.27) | 0.210 |
| Surgery for primary lesion (Yes vs. No) | 0.19 (0.07, 0.49) | <0.001 | 0.51 (0.25, 1.07) | 0.073 |
| Targeted therapy (Yes vs. No) | 0.53 (0.20, 1.42) | 0.198 | 2.82 (1.44, 5.51) | 0.003 |
| Patients with TP53 Mut (n=196) | | | | |
| Male (vs. Female) | 1.25 (0.73, 2.14) | 0.415 | 0.87 (0.60, 1.27) | 0.480 |
| Late onset (vs. Early onset) | 1.35 (0.66, 2.77) | 0.415 | 1.03 (0.65, 1.63) | 0.886 |
| KRAS Mut (vs. WT) | 1.39 (0.81, 2.38) | 0.237 | 1.51 (1.02, 2.22) | 0.039 |
| Right-sided (vs. Left-sided) | 1.60 (0.89, 2.86) | 0.111 | 0.90 (0.58, 1.40) | 0.655 |
| Stage IV (vs. Stage III) | 1.86 (1.00, 3.45) | 0.053 | 1.25 (0.81, 1.92) | 0.306 |
| Surgery for primary lesion (Yes vs. No) | 0.23 (0.13, 0.41) | <0.001 | 0.57 (0.37, 0.88) | 0.010 |
| Targeted therapy (Yes vs. No) | 0.56 (0.33, 0.95) | 0.032 | 2.15 (1.43, 3.23) | <0.001 |
| Patients with TP53 non-GOF Mut (n=65) | | | | |
| Male (vs. Female) | 0.80 (0.24, 2.65) | 0.721 | 0.45 (0.23, 0.89) | 0.022 |
| Late onset (vs. Early onset) | 1.20 (0.31, 4.66) | 0.793 | 2.70 (1.06, 6.88) | 0.036 |
| KRAS Mut (vs. WT) | 0.86 (0.24, 3.04) | 0.816 | 1.04 (0.50, 2.19) | 0.911 |
| Right-sided (vs. Left-sided) | 1.18 (0.32, 4.29) | 0.804 | 0.58 (0.25, 1.34) | 0.200 |
| Stage IV (vs. Stage III) | 4.39 (1.20, 16.01) | 0.021 | 1.68 (0.82, 3.44) | 0.154 |
| Surgery for primary lesion (Yes vs. No) | 0.39 (0.11, 1.39) | 0.142 | 0.71 (0.30, 1.67) | 0.436 |
| Targeted therapy (Yes vs. No) | 1.68 (0.50, 5.63) | 0.403 | 6.31 (2.76, 14.45) | <0.001 |
| Patients with TP53 GOF Mut (n=131) | | | | |
| Male (vs. Female) | 1.45 (0.76, 2.75) | 0.259 | 1.09 (0.69, 1.75) | 0.703 |
| Late onset (vs. Early onset) | 1.32 (0.53, 3.28) | 0.551 | 0.76 (0.43, 1.33) | 0.337 |
| KRAS Mut (vs. WT) | 1.42 (0.75, 2.66) | 0.284 | 1.72 (1.07, 2.78) | 0.026 |
| Right-sided (vs. Left-sided) | 1.91 (0.97, 3.79) | 0.058 | 1.15 (0.67, 1.97) | 0.615 |
| Stage IV (vs. Stage III) | 1.63 (0.78, 3.37) | 0.197 | 1.23 (0.72, 2.11) | 0.455 |
| Surgery for primary lesion (Yes vs. No) | 0.21 (0.10, 0.41) | <0.001 | 0.50 (0.29, 0.84) | 0.007 |
| Targeted therapy (Yes vs. No) | 0.40 (0.21, 0.76) | 0.005 | 1.64 (1.00, 2.69) | 0.050 |

**Supplementary Table 3.** **Parametric models for survival analysis by statuses of p53 Mutation based on LOF classification (N=247)**

|  | OS | | PFS | |
| --- | --- | --- | --- | --- |
|  | **HR (95%CI)** | **P value** | **HR (95%CI)** | **P value** |
| All patients (N=247) |  |  |  |  |
| Male (vs. Female) | 1.05 (0.64, 1.73) | 0.831 | 1.09 (0.77, 1.53) | 0.629 |
| Late onset (vs. Early onset) | 1.89 (1.00, 3.57) | 0.049 | 1.00 (0.68, 1.45) | 0.983 |
| KRAS Mut (vs. WT) | 1.44 (0.88, 2.36) | 0.144 | 1.62 (1.15, 2.28) | 0.005 |
| TP53 Mut (vs. WT) | 1.02 (0.61, 1.69) | 0.952 | 1.33 (0.94, 1.87) | 0.105 |
| Right-sided (vs. Left-sided) | 1.11 (0.63, 1.94) | 0.724 | 0.89 (0.60, 1.33) | 0.576 |
| Stage IV (vs. Stage III) | 1.70 (0.98, 2.94) | 0.056 | 1.11 (0.76, 1.63) | 0.576 |
| Surgery for primary lesion (Yes vs. No) | 0.20 (0.12, 0.36) | <0.001 | 0.62 (0.41, 0.93) | 0.020 |
| Targeted therapy (Yes vs. No) | 0.55 (0.34, 0.91) | 0.017 | 2.34 (1.62, 3.38) | <0.001 |
| Patients with WT TP53 (n=90) | | | | |
| Male (vs. Female) | 1.19 (0.50, 2.87) | 0.690 | 1.90 (1.06, 3.40) | 0.028 |
| Late onset (vs. Early onset) | 4.25 (1.26, 14.35) | 0.019 | 1.01 (0.54, 1.89) | 0.977 |
| KRAS Mut (vs. WT) | 1.31 (0.54, 3.13) | 0.545 | 1.09 (0.61, 1.96) | 0.760 |
| Right-sided (vs. Left-sided) | 1.10 (0.45, 2.68) | 0.834 | 1.39 (0.73, 2.65) | 0.310 |
| Stage IV (vs. Stage III) | 1.73 (0.64, 4.74) | 0.277 | 0.64 (0.33, 1.27) | 0.210 |
| Surgery for primary lesion (Yes vs. No) | 0.19 (0.07, 0.49) | <0.001 | 0.51 (0.25, 1.07) | 0.073 |
| Targeted therapy (Yes vs. No) | 0.53 (0.20, 1.42) | 0.198 | 2.82 (1.44, 5.51) | 0.003 |
| Patients with TP53 Mut (n=157) | | | | |
| Male (vs. Female) | 1.06 (0.57, 1.98) | 0.857 | 0.82 (0.54, 1.25) | 0.357 |
| Late onset (vs. Early onset) | 1.08 (0.49, 2.38) | 0.851 | 1.03 (0.62, 1.71) | 0.915 |
| KRAS Mut (vs. WT) | 1.73 (0.91, 3.30) | 0.094 | 1.92 (1.22, 3.02) | 0.005 |
| Right-sided (vs. Left-sided) | 1.11 (0.51, 2.41) | 0.788 | 0.72 (0.41, 1.26) | 0.253 |
| Stage IV (vs. Stage III) | 1.77 (0.88, 3.57) | 0.109 | 1.48 (0.93, 2.38) | 0.101 |
| Surgery for primary lesion (Yes vs. No) | 0.21 (0.10, 0.44) | <0.001 | 0.61 (0.36, 1.02) | 0.056 |
| Targeted therapy (Yes vs. No) | 0.47 (0.25, 0.88) | 0.016 | 2.10 (1.33, 3.32) | 0.002 |
| Patients with TP53 likely LOF Mut (n=98) | | | | |
| Male (vs. Female) | 0.95 (0.38, 2.35) | 0.910 | 0.71 (0.41, 1.21) | 0.207 |
| Late onset (vs. Early onset) | 1.93 (0.62, 5.98) | 0.246 | 1.19 (0.63, 2.25) | 0.601 |
| KRAS Mut (vs. WT) | 1.14 (0.45, 2.87) | 0.786 | 1.95 (1.10, 3.47) | 0.023 |
| Right-sided (vs. Left-sided) | 1.39 (0.53, 3.64) | 0.500 | 0.73 (0.38, 1.43) | 0.363 |
| Stage IV (vs. Stage III) | 2.99 (1.04, 8.56) | 0.045 | 1.86 (1.02, 3.39) | 0.042 |
| Surgery for primary lesion (Yes vs. No) | 0.28 (0.11, 0.72) | 0.005 | 0.58 (0.31, 1.09) | 0.087 |
| Targeted therapy (Yes vs. No) | 0.90 (0.34, 2.39) | 0.837 | 2.16 (1.15, 4.05) | 0.019 |
| Patients with TP53 known LOF Mut (n=59) | | | | |
| Male (vs. Female) | 1.23 (0.47, 3.18) | 0.672 | 0.97 (0.48, 1.97) | 0.933 |
| Late onset (vs. Early onset) | 0.91 (0.28, 2.95) | 0.875 | 0.84 (0.36, 1.97) | 0.686 |
| KRAS Mut (vs. WT) | 3.79 (1.40, 10.29) | 0.010 | 1.99 (0.94, 4.22) | 0.075 |
| Right-sided (vs. Left-sided) | 0.60 (0.13, 2.68) | 0.507 | 0.65 (0.22, 1.91) | 0.441 |
| Stage IV (vs. Stage III) | 0.43 (0.11, 1.64) | 0.220 | 1.16 (0.49, 2.75) | 0.728 |
| Surgery for primary lesion (Yes vs. No) | 0.05 (0.01, 0.29) | <0.001 | 0.68 (0.24, 1.90) | 0.453 |
| Targeted therapy (Yes vs. No) | 0.21 (0.07, 0.60) | 0.002 | 2.07 (0.97, 4.41) | 0.063 |
