# Supplementary figures and images for: The heterogeneous impact of targeted therapy on the prognosis of stage III/IV colorectal cancer patients with different subtypes of TP53 mutations
Source: Cancer Med. 2023 Dec 8;12(24):21920–32. doi: 10.1002/cam4.6766 (PMC10757131; doi:10.1002/cam4.6766)

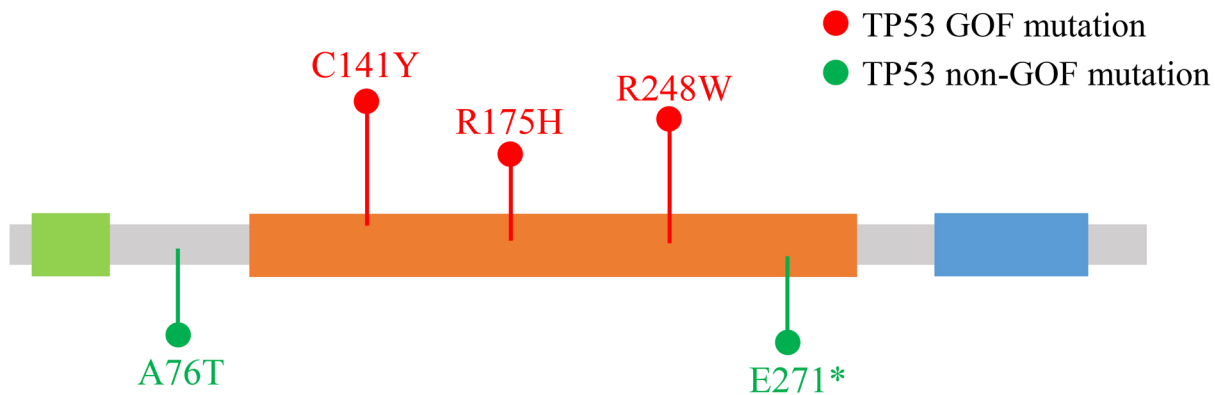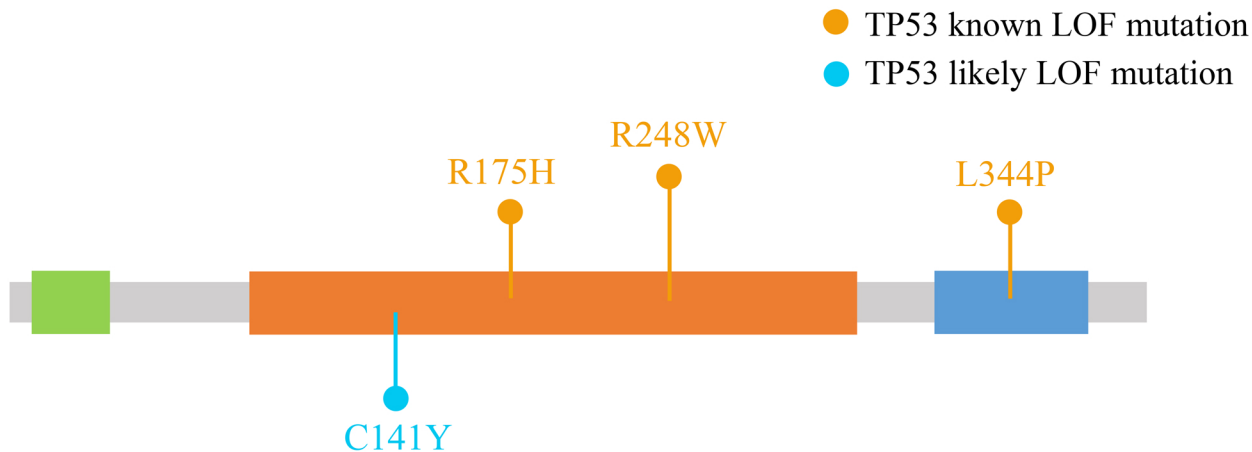

- P53\_TAD: P53 transactivation motif (6 - 29)
- P53: P53 DNA-binding domain (95 - 288)
- P53\_tetramer: P53 tetramerisation motif (318 - 358)

Supplement: Supplementary file 3 — Figure S1. [file CAM4-12-21920-s003.pdf]

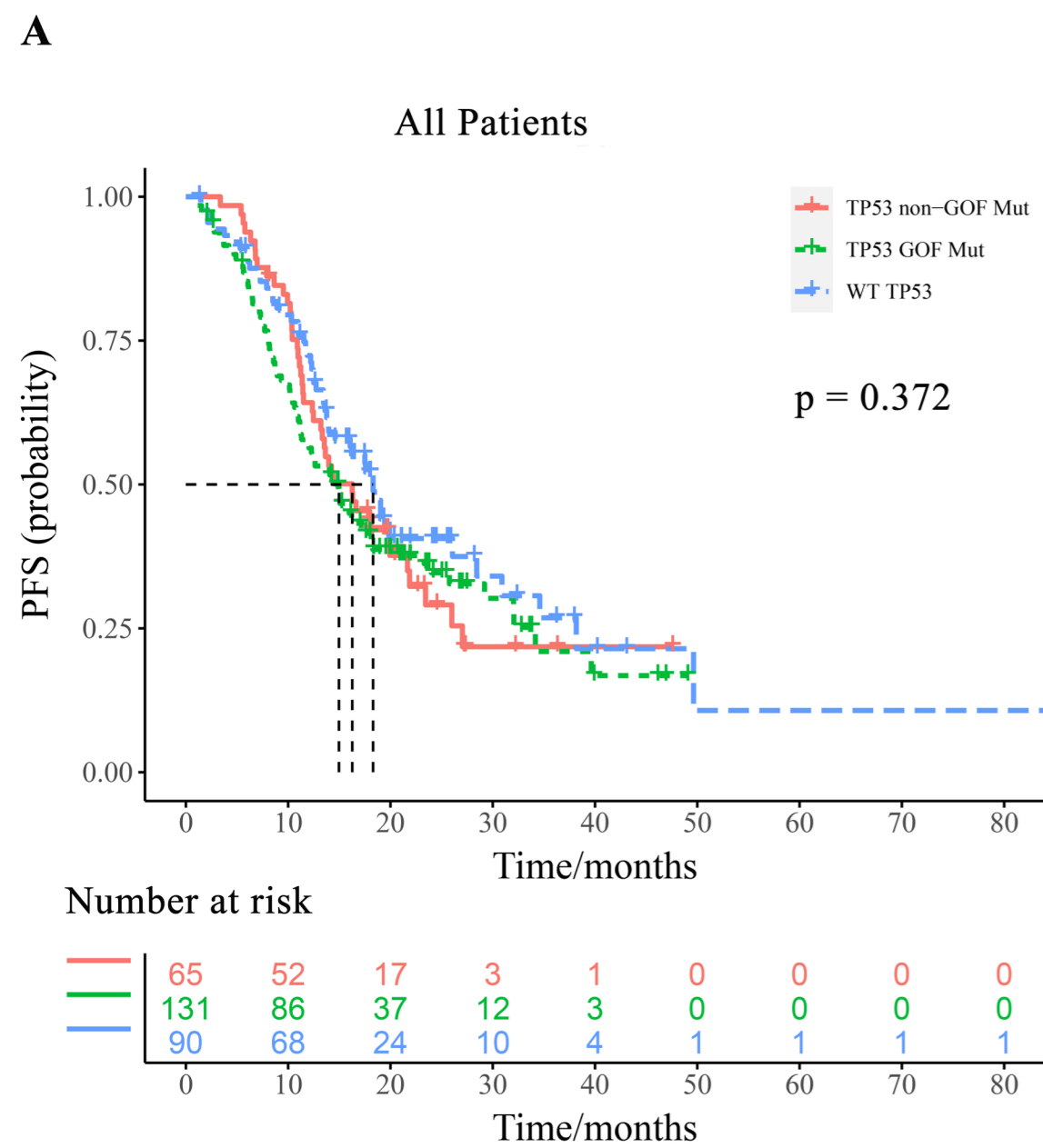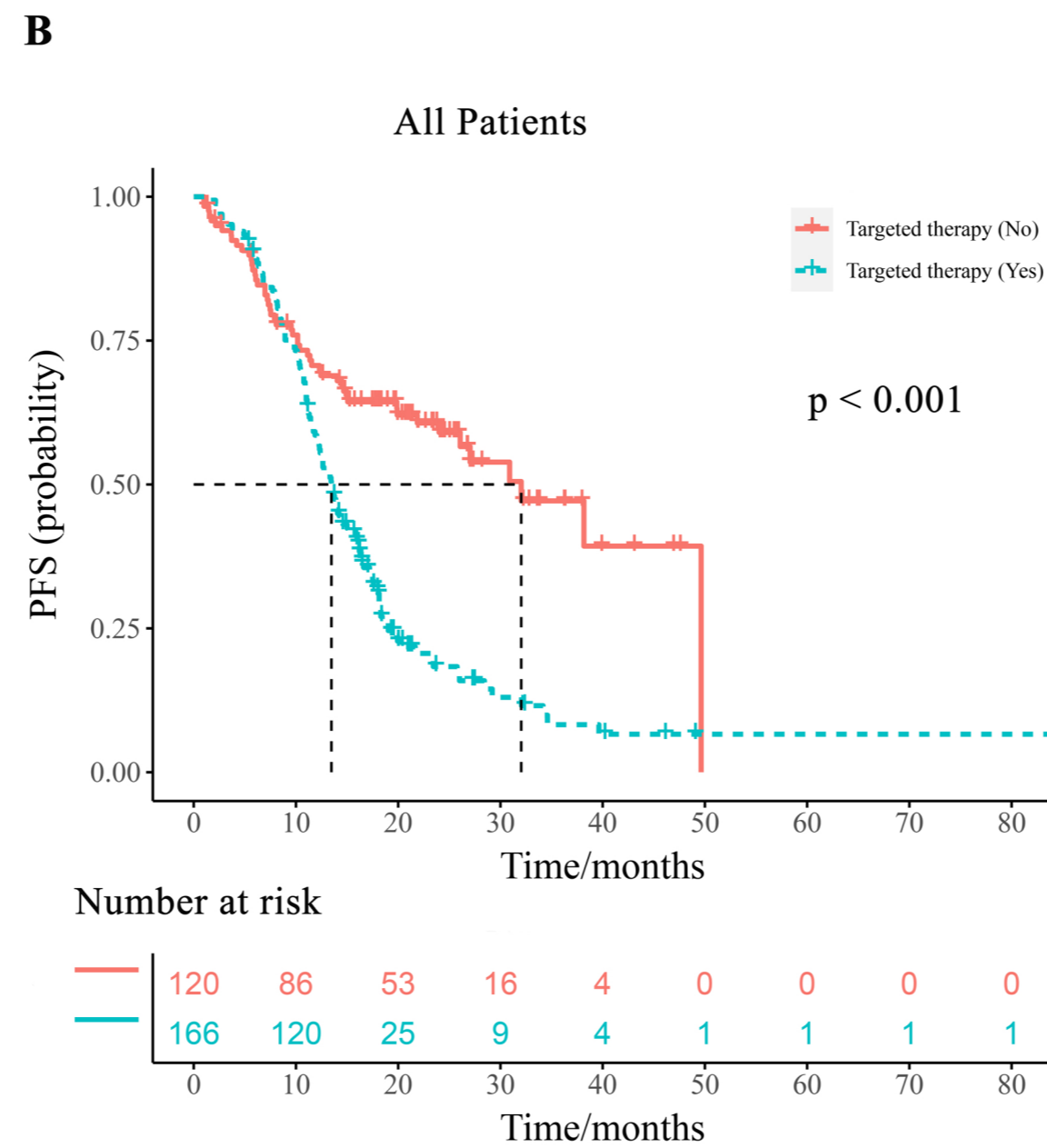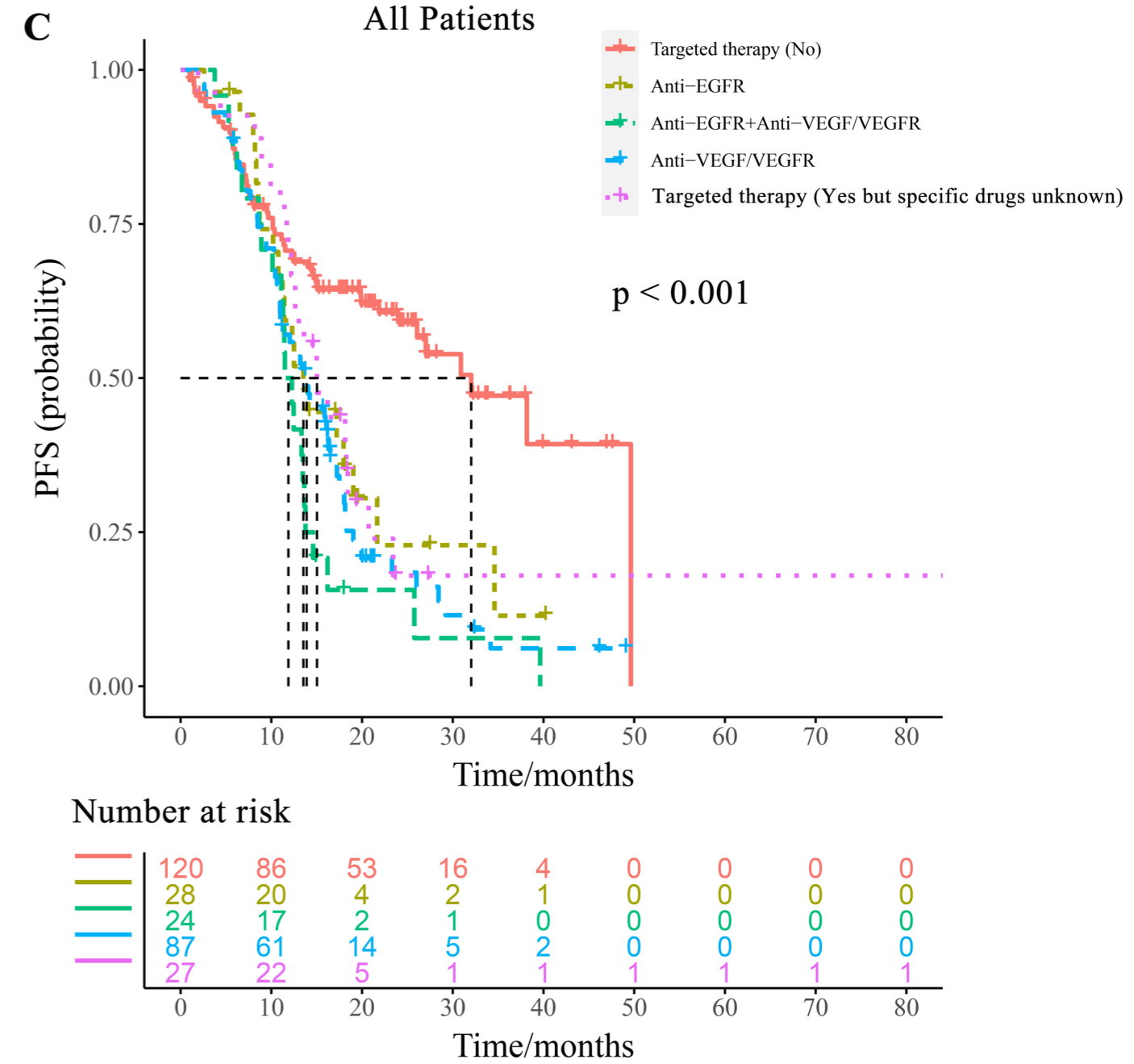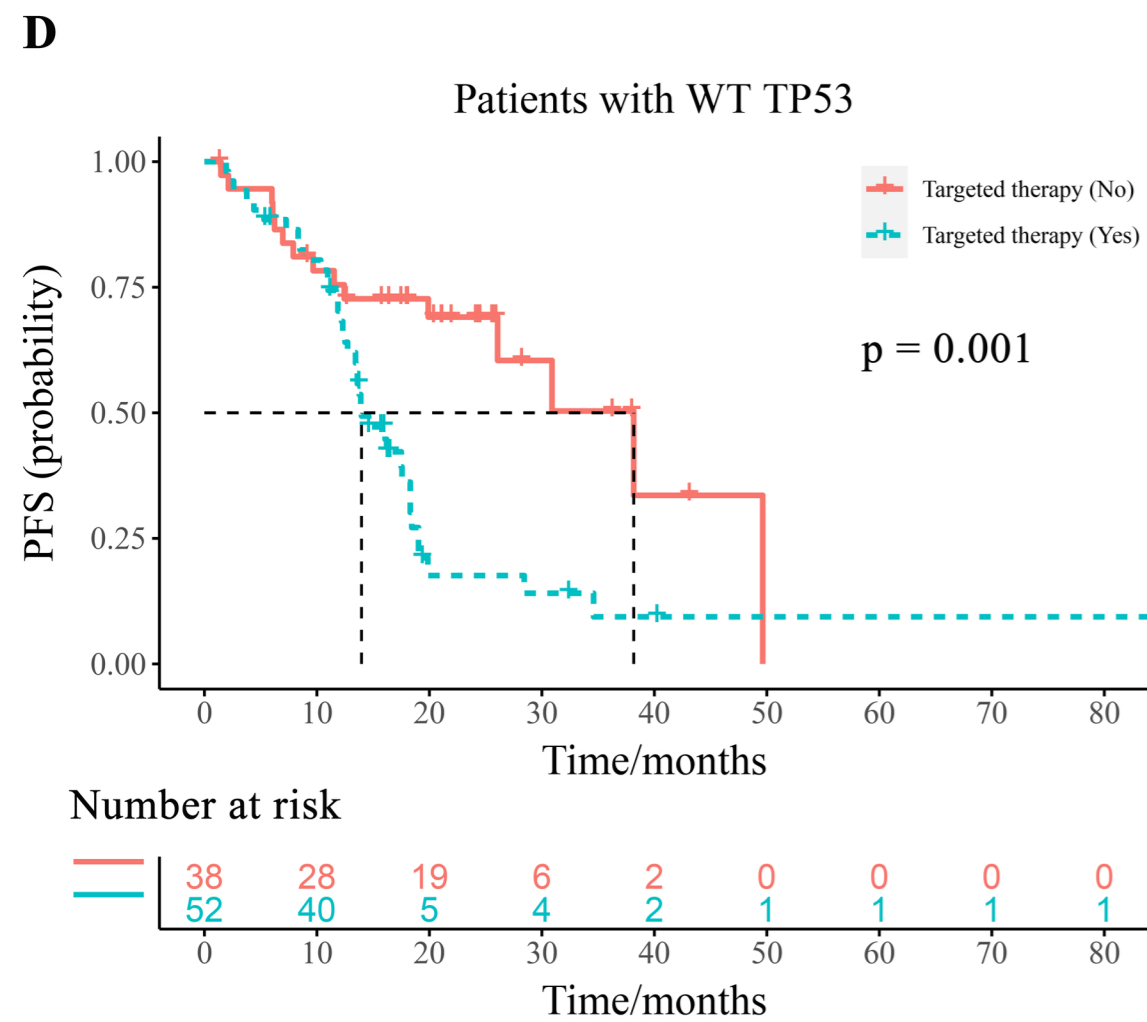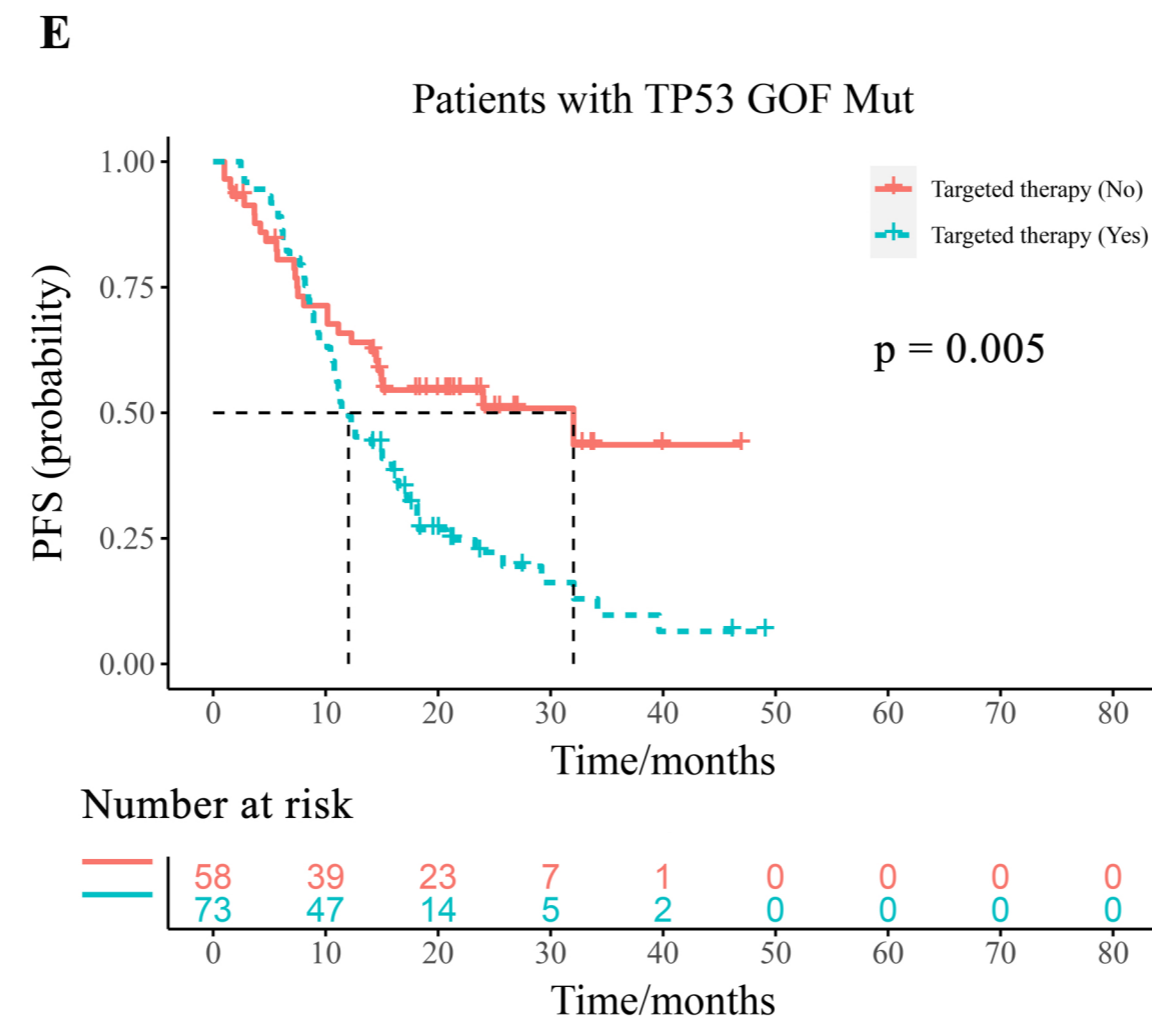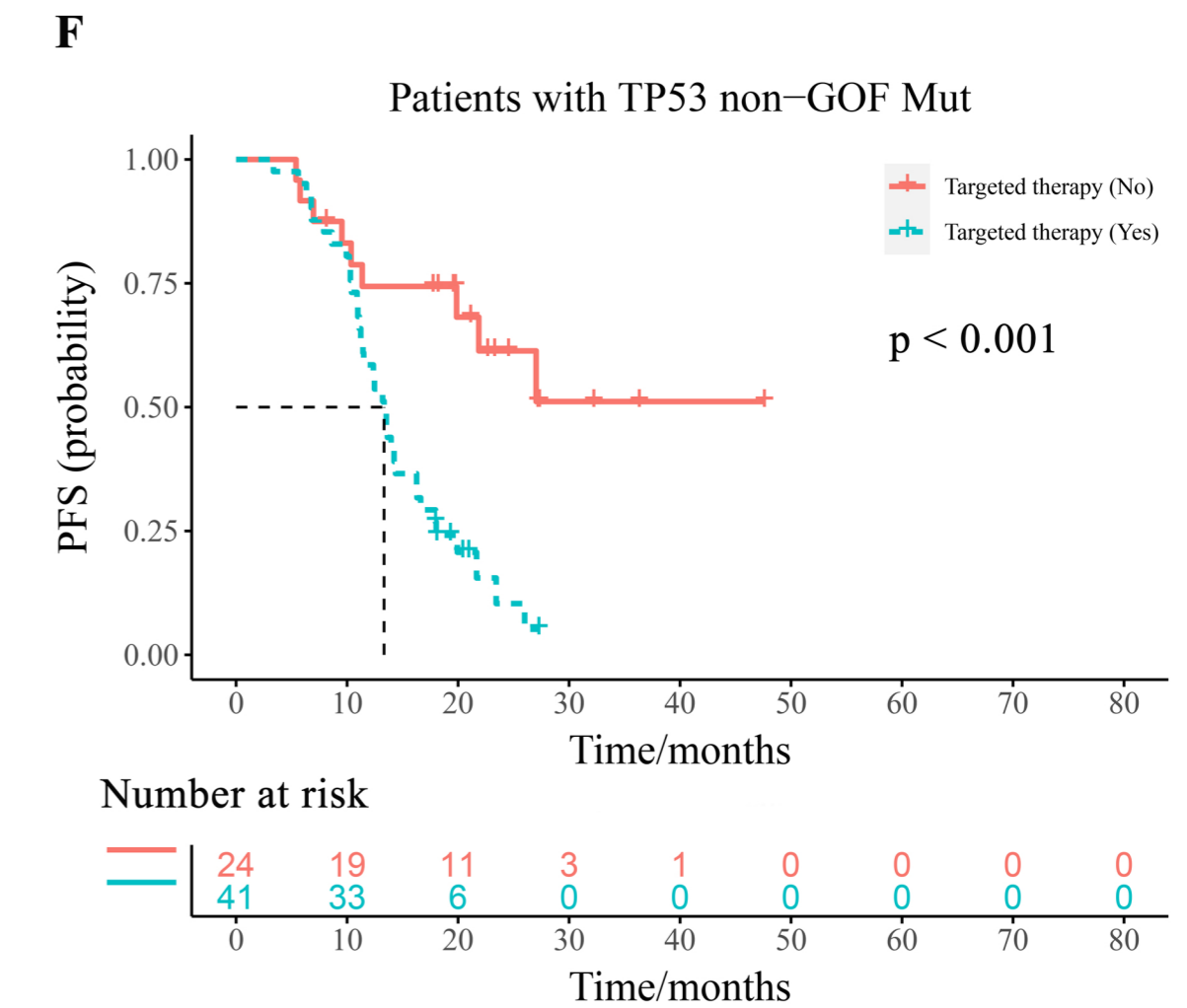

Supplement: Supplementary file 4 — Figure S2. [file CAM4-12-21920-s004.pdf]

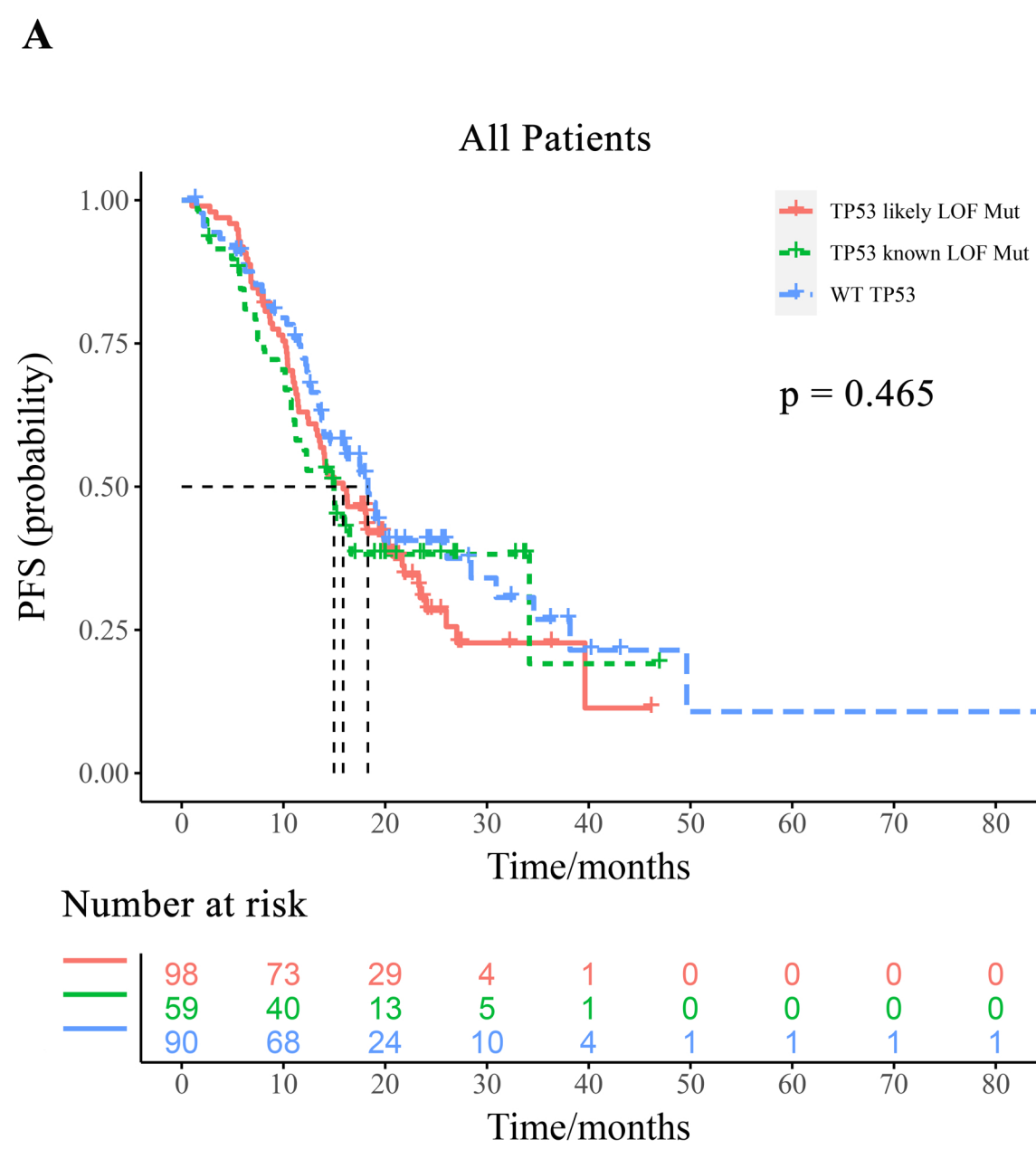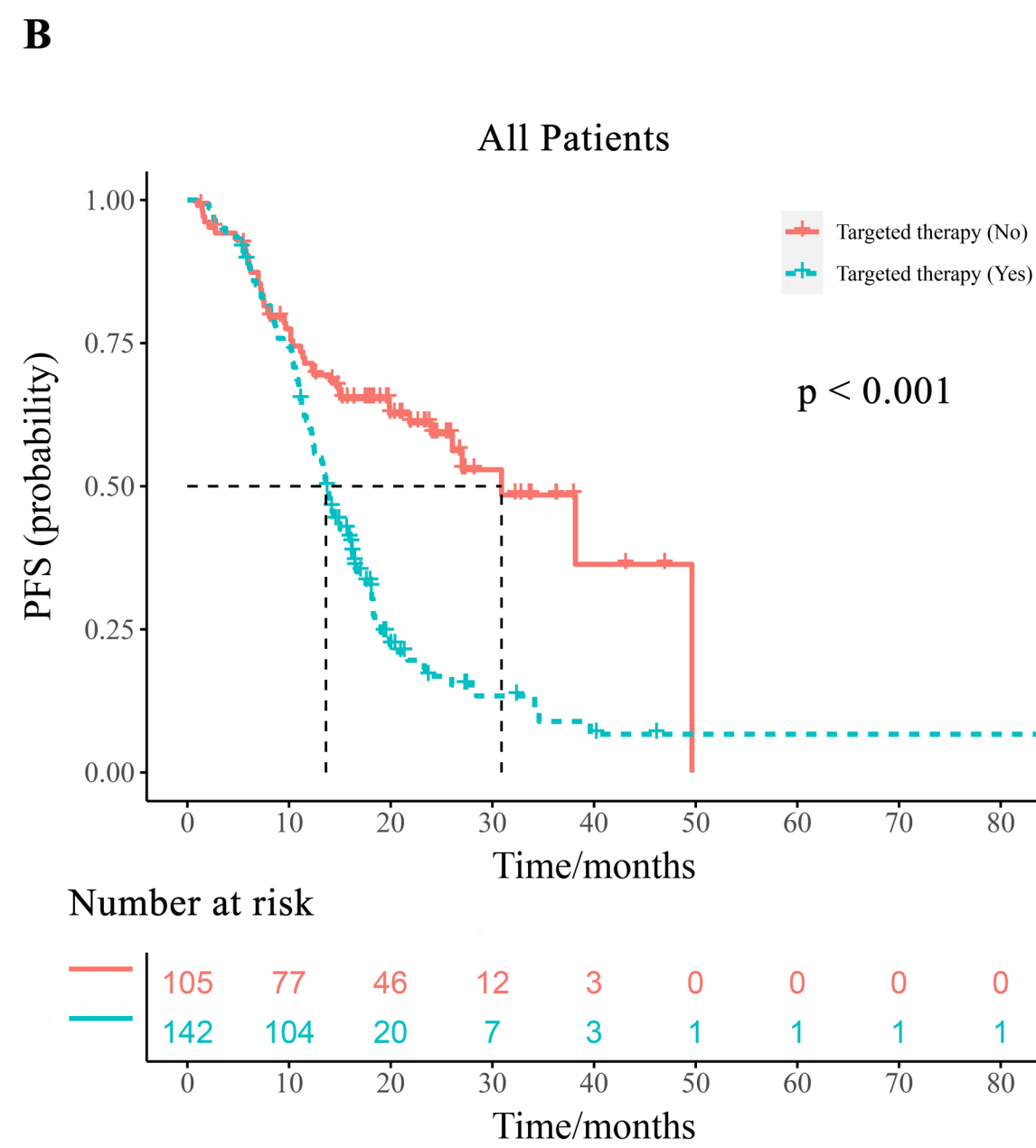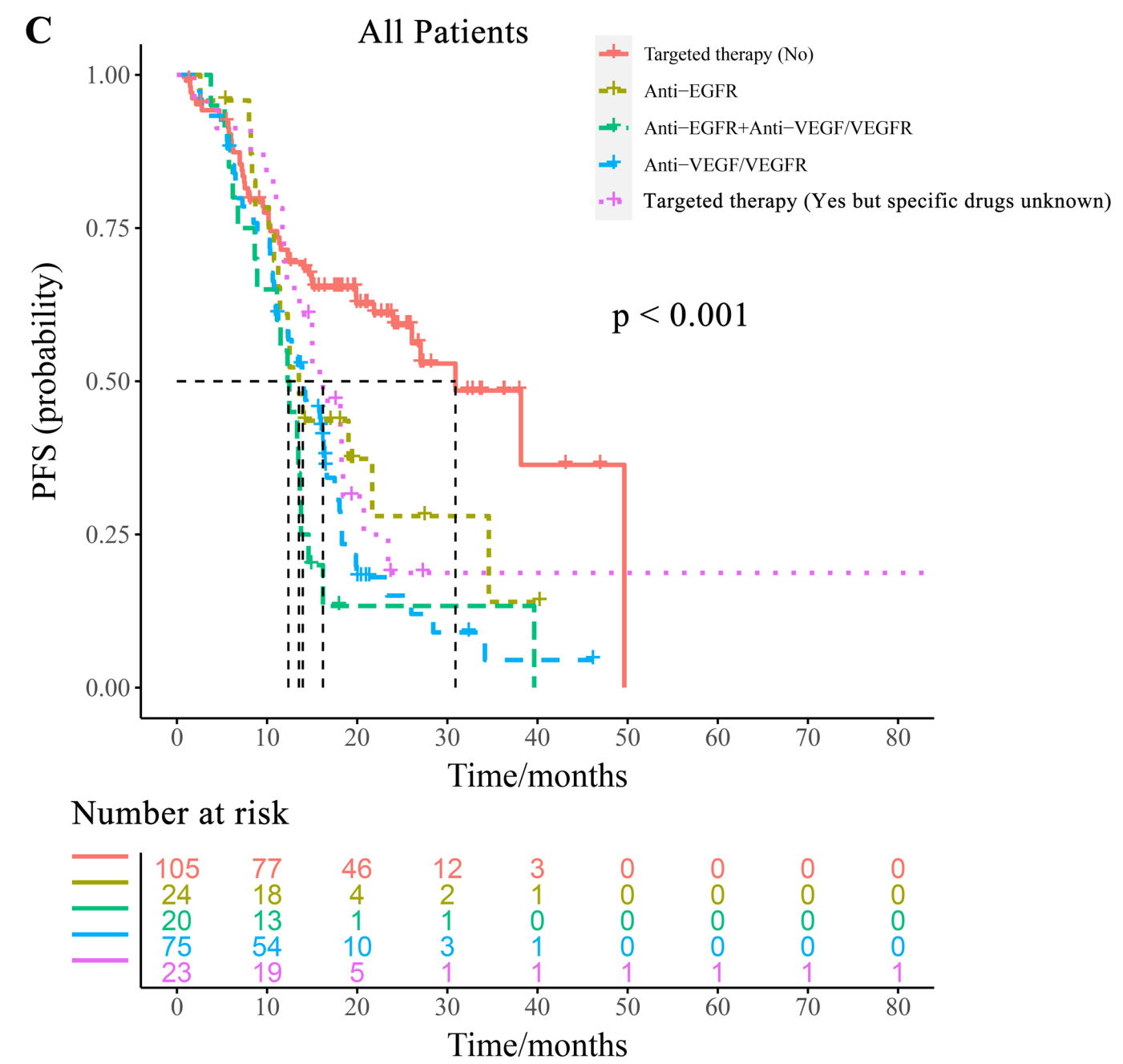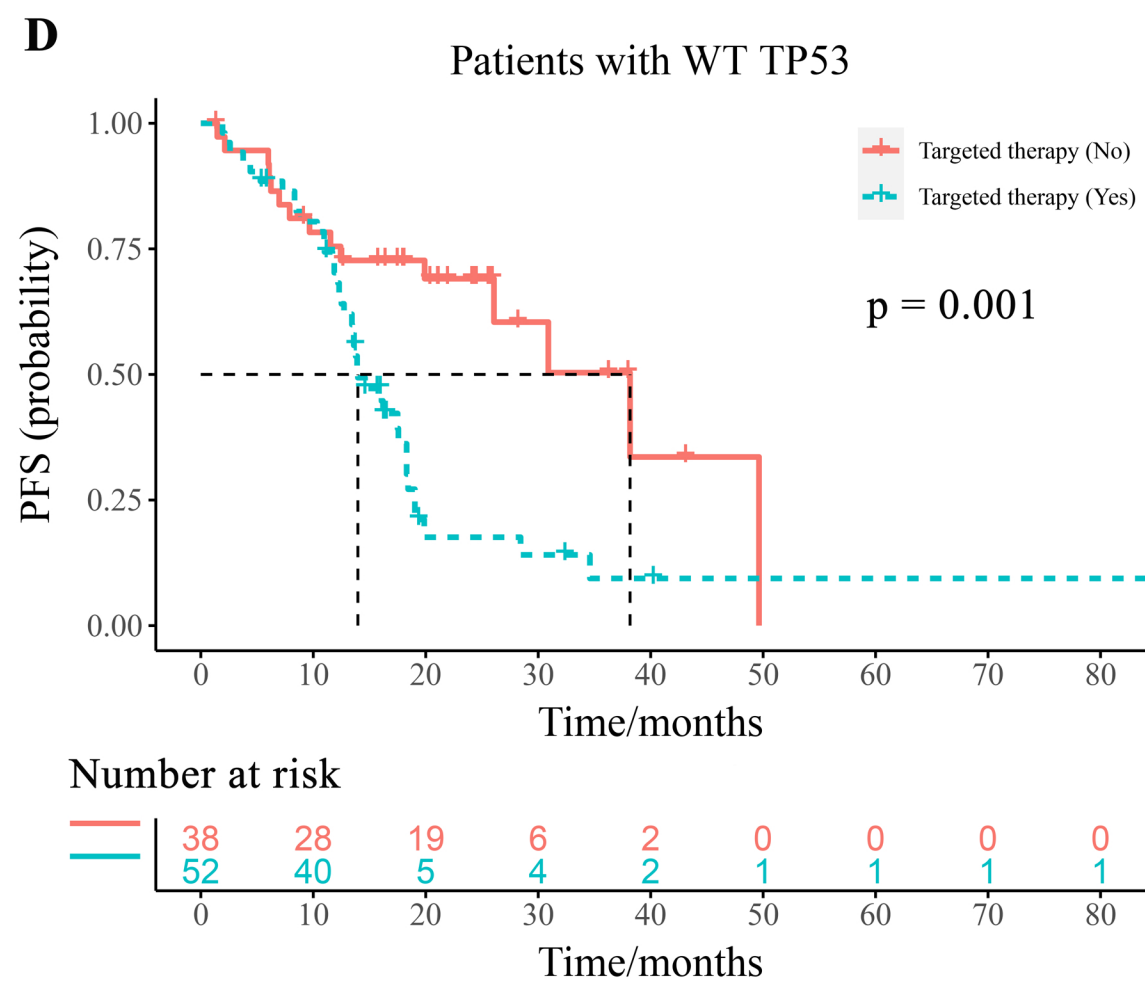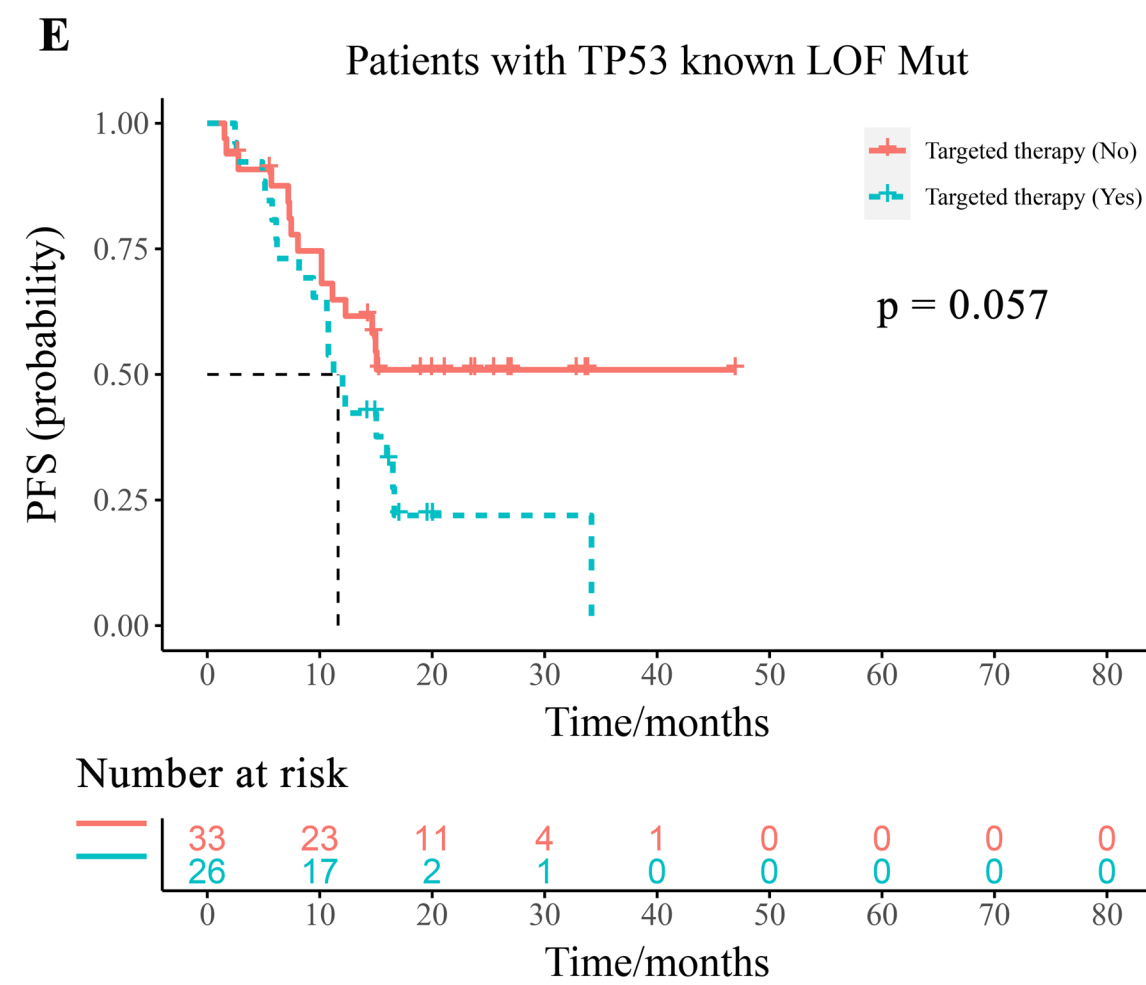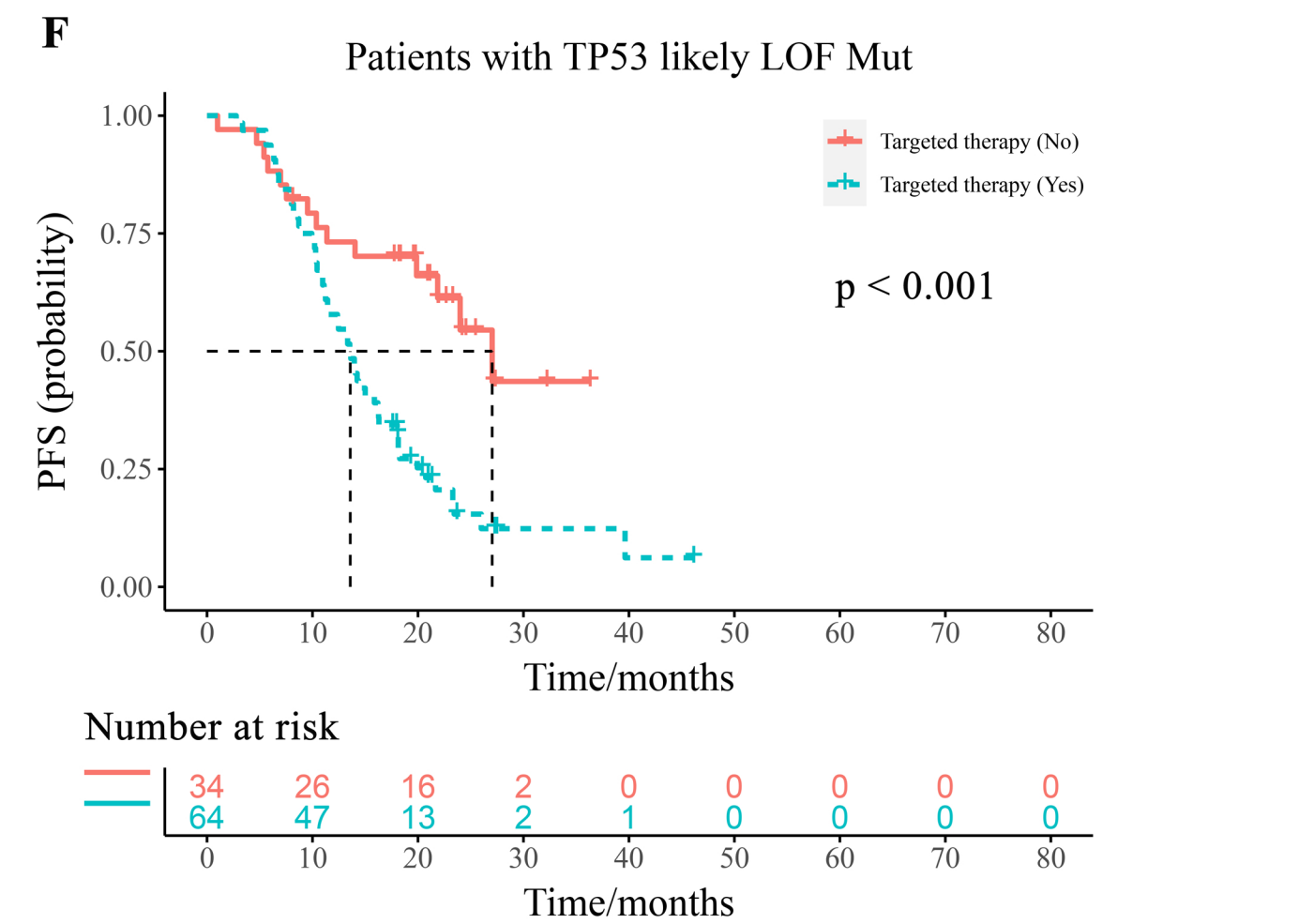

Supplement: Supplementary file 5 — Figure S3. [file CAM4-12-21920-s006.pdf]
