# Supplementary material for: The heterogeneous impact of targeted therapy on the prognosis of stage III/IV colorectal cancer patients with different subtypes of TP53 mutations
Source: Cancer Med. 2023 Dec 8;12(24):21920–32. doi: 10.1002/cam4.6766 (PMC10757131; doi:10.1002/cam4.6766)
Supplement: Supplementary file 6 — Data S2. [file CAM4-12-21920-s001.docx]

**Supplementary Figure 1**

Pattern diagram of TP53 GOF/non-GOF mutations and known/likely LOF mutations.

**Supplementary Figure 2**

(2A) to (2F), progression-free survival (PFS) of patients with different statuses of TP53 mutations and targeted therapy in TP53 mutation GOF classification set.

**Supplementary Figure 3**

(3A) to (3F), progression-free survival (PFS) of patients with different statuses of TP53 mutations and targeted therapy in TP53 mutation LOF classification set.
